# Independent expansion, selection, and hypervariability of the *TBC1D3* gene family in humans

**DOI:** 10.1101/gr.279299.124

**Published:** 2024-11

**Authors:** Xavi Guitart, David Porubsky, DongAhn Yoo, Max L. Dougherty, Philip C. Dishuck, Katherine M. Munson, Alexandra P. Lewis, Kendra Hoekzema, Jordan Knuth, Stephen Chang, Tomi Pastinen, Evan E. Eichler

**Affiliations:** 1 Department of Genome Sciences, University of Washington School of Medicine, Seattle, Washington 98195, USA;; 2 Tisch Cancer Institute, Division of Hematology and Medical Oncology, The Icahn School of Medicine at Mount Sinai, New York, New York 10029, USA;; 3 Department of Biochemistry;; 4 Department of Medicine, Division of Cardiovascular Medicine, Stanford University, Stanford, California 94305, USA;; 5 Department of Pediatrics, Genomic Medicine Center, Children's Mercy Kansas City, Kansas City, Missouri 64108, USA;; 6 Department of Pediatrics, School of Medicine, University of Missouri Kansas City, Kansas City, Missouri 64108, USA;; 7 Howard Hughes Medical Institute, University of Washington, Seattle, Washington 98195, USA

## Abstract

*TBC1D3* is a primate-specific gene family that has expanded in the human lineage and has been implicated in neuronal progenitor proliferation and expansion of the frontal cortex. The gene family and its expression have been challenging to investigate because it is embedded in high-identity and highly variable segmental duplications. We sequenced and assembled the gene family using long-read sequencing data from 34 humans and 11 nonhuman primate species. Our analysis shows that this particular gene family has independently duplicated in at least five primate lineages, and the duplicated loci are enriched at sites of large-scale chromosomal rearrangements on Chromosome 17. We find that all human copy-number variation maps to two distinct clusters located at Chromosome 17q12 and that humans are highly structurally variable at this locus, differing by as many as 20 copies and ∼1 Mbp in length depending on haplotypes. We also show evidence of positive selection, as well as a significant change in the predicted human TBC1D3 protein sequence. Last, we find that, despite multiple duplications, human *TBC1D3* expression is limited to a subset of copies and, most notably, from a single paralog group: *TBC1D3-CDKL*. These observations may help explain why a gene potentially important in cortical development can be so variable in the human population.

Gene duplication followed by adaptation is one of the primary forces by which new genes emerge within species (Ohno 1970). Many of these evolutionary events occur in segmental duplications (SDs), genomic units that are at least 1 kbp in length and whose duplications are ≥90% identical to one another (Bailey and Eichler 2006). Many human-specific genes reside in SDs, which often continue to vary structurally in our lineage (Bitar et al. 2019). Since the initial publication of the human and chimpanzee genomes, investigations of human-specific SD genes have found that they most often are implicated in xenobiotic recognition, metabolism, immunity, and neuronal development, playing an important role in the evolution of our species (Perry et al. 2007; Dennis et al. 2012; Huttner et al. 2024).

*TBC1D3* is a primate-specific SD gene family (Paulding et al. 2003). This gene family is dispersed across the two arms of Chromosome 17, although most copies in humans map to two expansion blocks at locus Chromosome 17q12 (Fig. 1A). Expression data in humans from the Genome-Tissue Expression (GTEx) project reveal *TBC1D3* is modestly expressed globally, with increased expression in testis and brain tissue (The GTEx Consortium 2020). *TBC1D3* expression and function were initially observed in prostate tumor samples and originally classified as an oncogene (Hodzic et al. 2006). However, in 2016, Ju et al. showed that transgenic overexpression of *TBC1D3* in the developing mouse brain results in a proliferation of outer radial glial cells and a subsequent expansion and folding of the cortex (Ju et al. 2016).

**Figure 1.**
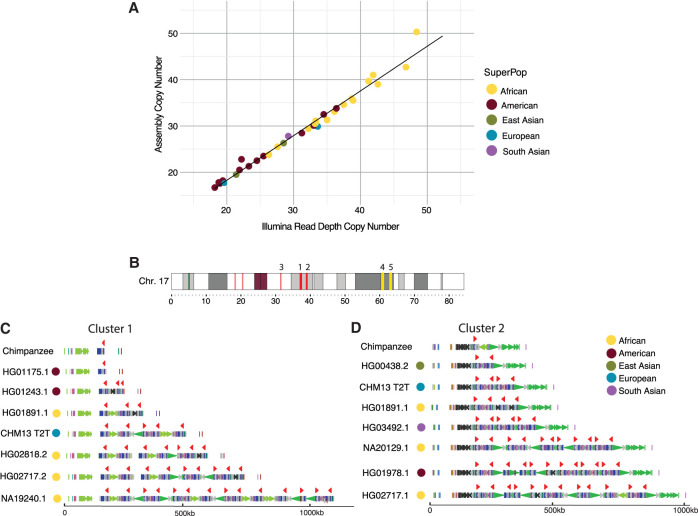
Assembly and human variation of *TBC1D3.* (*A*) Assembly copy-number estimate versus orthogonal Illumina sequence copy-number estimate. Each point represents a sample diploid assembly, colored by superpopulation. (*B*) Reference ideogram of *TBC1D3* regions. Expanded views of clusters 1 and 2 (marked in red) are illustrated in *C*,*D*. (*C*,*D*) Structure for chimpanzee and seven validated human haplotypes over *TBC1D3* cluster 1 (*C*) and cluster 2 (*D*). *TBC1D3* copies are colored as red arrows. Colored arrows *below TBC1D3* illustrate segmental duplication content annotated with DupMasker (Jiang et al. 2008).

These findings suggest that the evolution of *TBC1D3* may have contributed to human cranial expansion over the past two million years (Stringer 2016). Investigations of the sequence evolution and variation among humans and nonhuman primates (NHPs) would help test this hypothesis (Sabeti et al. 2006). However, the duplicated and highly identical sequences of *TBC1D3* copies make assembly impossible with standard short-read sequencing platforms. Instead, researchers have investigated copy-number variation in SD genes using short-read sequencing data to understand patterns of variation (Sudmant et al. 2010). Such read-based studies have suggested extensive copy-number differences among human populations. However, these experiments lack the single-base-pair resolution necessary to distinguish different paralogous copies, structural differences among haplotypes, and which copies are likely functional or expressed. Moreover, it is unclear how a gene so variable in copy number could play such a critical role in the expansion of the frontal cortex in humans. In this study, we address these questions by leveraging long-read sequencing data generated from humans and apes to fully resolve the *TBC1D3* loci (Liao et al. 2023; Makova et al. 2024; Mao et al. 2024). The goals of this study were to reconstruct the evolutionary history of this gene family, to assess the extent of human genetic diversity, and to determine how this variation relates to changes in selection and expression of the gene family in the human lineage.

## Results

### Human *TBC1D3* copy-number variation

To understand *TBC1D3* organization and variation in humans, we first focused on two *TBC1D3* gene family clusters, named cluster 1 and cluster 2, that contain the majority of *TBC1D3* paralogs (Fig. 1B). We characterized 44 human genomes recently sequenced as part of the Human Pangenome Reference Consortium at this locus (Supplemental Table S1; Liao et al. 2023). We first assessed the integrity of each assembly by searching for sequence collapses in read depth of both Pacific Biosciences (PacBio) high-fidelity (HiFi) and Oxford Nanopore Technologies (ONT) sequencing data (Methods) (Supplemental Table S2; Vollger et al. 2019; Dishuck et al. 2023). We found that 46 of the haplotypes passed quality control (QC), whereas 42 haplotypes failed. We attempted to reassemble the samples that failed QC using a novel assembly algorithm that leverages both HiFi and ONT data (Verkko) (Rautiainen et al. 2023). This procedure recovered an additional 20 haplotypes in which both cluster 1 and cluster 2 were fully sequenced and assembled without error (Supplemental Fig. S1). We also confirmed accurate assembly with an orthogonal sequencing platform by comparing assembly-predicted against Illumina read depth–based copy-number estimates (Methods) (Fig. 1A; Supplemental Fig. S2). For our investigations, we required that both haplotypes of the assembly accurately resolve. In total, we validated 66 haplotypes in which both *TBC1D3* clusters were fully resolved and, including three genome references, developed a total data set of 69 human haplotypes.

Next, we estimated the copy number and organization of *TBC1D3* in clusters 1 and 2 for each human haplotype (Fig. 1B–D). In cluster 1, we found that *TBC1D3* varies from one to 14 copies, whereas in cluster 2, it varies from two to 14 copies (Supplemental Table S3). Thus, the human diplotype copy number for *TBC1D3* summing across both clusters could theoretically range from six to 56 based on our limited survey of human diversity. The differences in copy account for as much as 1.5 Mbp of the differential size between human haplotypes. Notably, we find that the *TBC1D3* copy number is significantly higher among African (*X* = 34.4) compared with non-African populations (*X* = 25.4; *P*-value = 1.7 × 10^−5^). Higher African copy number is an observation that has been confirmed by Illumina whole-genome sequencing (WGS) read-depth analysis for *TBC1D3* and seen for other recently duplicated copy-number-polymorphic loci (Vollger et al. 2022; Jeong et al. 2024). The basis for this is unknown, but it may reflect the genetic bottleneck in the out-of-African founder populations or another manifestation of overall increased genetic diversity of African populations. For cluster 1, we find that 65% (45/69) of the haplotypes are structurally distinct. Additionally, for cluster 2, we observe similar diversity, in which 68% (47/69) are structurally distinct (Supplemental Fig. S3). Based on completely assembled diploid samples, we estimate the structural heterozygosity for cluster 1 is 94% and for cluster 2 is 88%, making these two loci among some of the most structurally variable gene families in the human genome (Sudmant et al. 2010).

### NHP *TBC1D3* organization

To better understand the evolution of the clusters, we investigated the organization of *TBC1D3* in 10 different NHP lineages (Supplemental Table S4). This included single representatives of five great ape species (bonobo, chimpanzee, gorilla, Bornean, and Sumatran orangutan), two Old World monkeys (macaque and gelada), two New World monkeys (marmoset and owl monkey), and one prosimian (mouse lemur). Eight of these genomes were previously published (Mao et al. 2024) or are part of efforts to generate telomere-to-telomere (T2T) assemblies of ape genomes (Makova et al. 2024). We generated HiFi sequence data from both the gelada and mouse lemur genomes in this study and assembled their genomes using Hifiasm (Methods).

**Figure 2.**
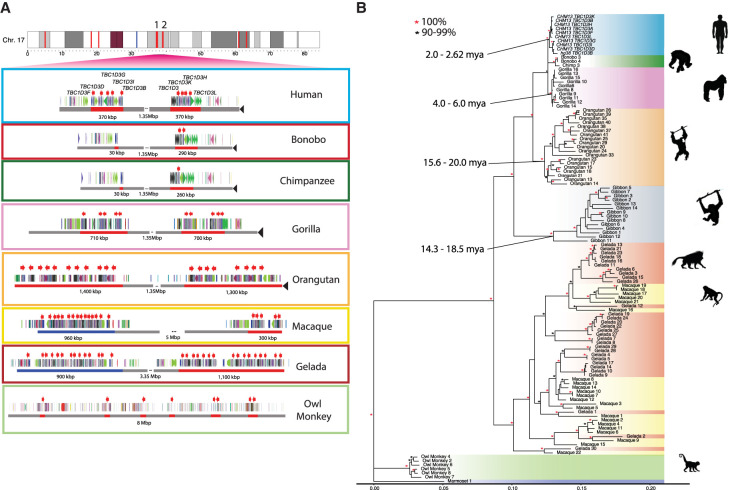
Comparative genome structure and phylogeny of *TBC1D3* gene family among primates. (*A*) *TBC1D3* clusters 1 and 2 structure. Orthologous *TBC1D3* clusters 1 and 2 are illustrated as two clustered regions (red blocks), with flanking unique sequence in gray for the primate lineages. Old World monkey *TBC1D3* expansion 1, which is nonsyntenic, is highlighted in blue. *TBC1D3* paralogs (red arrows) are embedded within other segmental duplication blocks, with DupMasker annotations illustrated with colored arrows. The diverse organizational differences of each expansion, including expansion size, duplicon content, and copy number, suggest independent expansion. (*B*) *TBC1D3* neutral phylogeny generated by maximum likelihood; 2300 bp of intronic sequence were aligned between all primate *TBC1D3* paralogs observed in *A*, with the marmoset sequence used as an outgroup. The phylogeny supports the hypothesis of independent expansion with the exception of the Old World monkeys (geladas and macaques) in which several copies duplicated before and after speciation of these two lineages (11 mya) (Liedigk et al. 2014).

With the exception of the mouse lemur, all NHP genomes carry multiple copies of *TBC1D3* (Supplemental Table S5). We find that *TBC1D3* is also highly copy-number-variable among NHPs, from two copies in the marmoset to 31 copies within a single haplotype in both the gelada and gibbon. We searched specifically for clustered expansions and found that most primates—human, gorilla, orangutan, macaque, and gelada—similarly contain two expanded clusters of *TBC1D3* (Fig. 2A). Among apes, these two clusters are orthologous to human clusters 1 and 2, separated by 1.35 Mbp of intervening sequence. Among the Old World monkeys, geladas and macaques, structural rearrangements have repositioned the two clusters such that the intervening sequence is larger and nonsyntenic. Importantly, bonobos and chimpanzees only possess one to two copies of *TBC1D3* at cluster 2, whereas no copies were identified at cluster 1. Thus, all humans have an increase in copy number compared with the *Pan* lineage but are not exceptional compared with most other NHP lineages. New World monkeys, owl monkeys, and marmosets do not have *TBC1D3* organized into clusters. Instead, the marmoset has two copies and the owl monkey has eight copies distributed throughout its chromosome, suggesting independent and recent expansions. Overall, we find that *TBC1D3* copy number varies from zero to 14 copies in cluster 1 and from one to 17 copies in cluster 2 (Fig. 2B). A detailed analysis of the composition of the SDs within each primate lineage shows that the units of duplication in different species frequently differed in structure, suggesting independent duplications or gene conversion events in each lineage (Methods) (Supplemental Fig. S4).

To estimate when the clustered *TBC1D3* copies expanded in each lineage, we constructed a maximum likelihood phylogenetic tree based on a multiple sequence alignment (MSA) generated from intronic sequence of each predicted *TBC1D3* gene copy from the various primate genomes (Methods) (Fig. 2B). We observe complete lineage-specific stratification of the *TBC1D3* gene family members into distinct clades for the human, *Pan*, gorilla, orangutan, gibbon, and owl monkey lineages. These findings strongly support recurrent duplication or gene conversion of all gene family copies in each lineage. In contrast, the gelada and rhesus macaque show both shared and lineage-specific groups, suggesting *TBC1D3* expanded before and after speciation. Using 25 and 6.5 million years ago (mya) as times of human–macaque and human–chimpanzee divergence, we estimated the timing of each lineage-specific expansion (Fig. 2B; Stevens et al. 2013). In most lineages, the primate duplications occurred relatively recently. Most notably, we observe that humans experienced the most recent expansion within the apes, occurring between 2.0 and 2.6 mya.

### *TBC1D3* and large-scale chromosomal rearrangements

**Figure 3.**
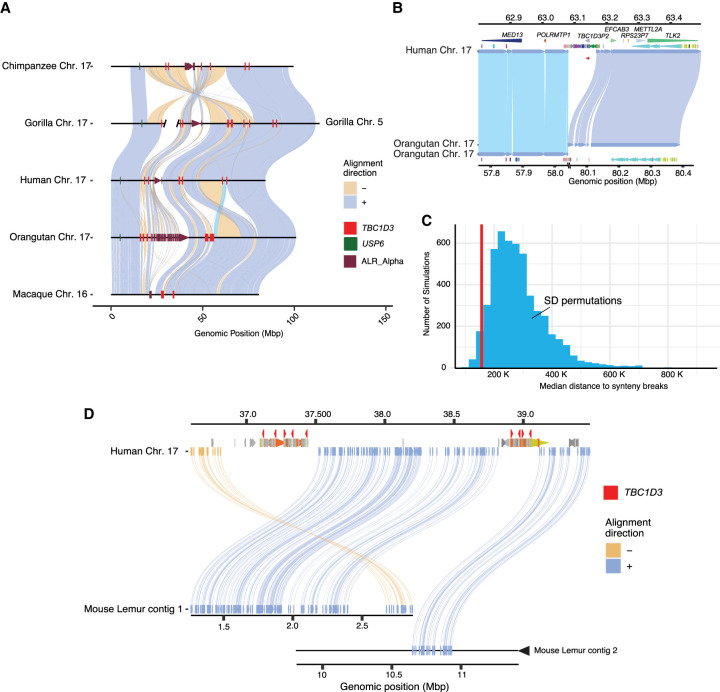
Large-scale chromosomal rearrangements and *TBC1D3* duplications. (*A*) Synteny plots of orthologous Chromosome 17 in primates reveal syntenic blocks in direct (blue) and inverted (yellow) orientation. Alpha satellite sequence, *TBC1D3* copies, and *USP6*—a hominoid fusion gene of *TBC1D3*—are illustrated in maroon, red, and green, respectively. *TBC1D3* demarcates the boundaries of large-scale rearrangements on chromosome phylogenetic group XVII. (*B*) *TBC1D3* duplication block (cluster of colored arrows) demarcates the boundary of a 12 Mbp inversion between the human and orangutan chromosomes. (*C*) Permutation test of segmental duplication proximity to synteny breaks. Five thousand permutation tests were performed, in which segmental duplication samples were taken, and median proximity to breaks in synteny was measured. True *TBC1D3* mappings fall within the lowest 3% of the permutations (red line), suggesting a nonrandom association between *TBC1D3* and breakpoints in synteny. (*D*) Synteny plot showing orthologous alignments between human *TBC1D3* and mouse lemur flanking genomic sequence.

During our comparative analysis of NHP genomes, we noticed that chromosomal synteny frequently was disrupted at sites corresponding to interspersed *TBC1D3* loci. To assess this more systematically, we selected five primate lineages for which T2T assemblies had recently been generated as part of the Primate T2T Consortium, aligned orthologous Chromosome 17s to one another, and illustrated these alignments, as well as alpha satellite and *TBC1D3* loci (Methods) (Fig. 3A). We found that *TBC1D3* consistently flanks some of the largest chromosomal rearrangements. For example, human *TBC1D3P2* demarcates one end of a 12 Mbp large-scale chromosomal inversion distinguishing human and Sumatran orangutan chromosomes (see light blue alignment in Fig. 3A,B). In the orangutan, the corresponding breakpoint of synteny is anchored in one of the expanded *TBC1D3* clusters. This structure is syntenic with the macaque, suggesting that it was the ancestral configuration, whereas the human structure, shared with gorillas and chimpanzees, was derived. Similarly, one of the fission breakpoints of Chromosome 17 resulting in gorilla Chromosomes 4 and 19 (Stankiewicz et al. 2001) maps precisely to *TBC1D3* and *USP6* duplications in the gorilla lineage.

To test if the association with *TBC1D3* and breakpoints of synteny was significant, we developed a permutation test. We randomly selected an equivalent sequence and number of mappings throughout Chromosome 17 for these five orthologous primate chromosomes and measured the median distance of these mappings to the nearest synteny break. In more than 5000 permutation tests, we never observed a distance as low as that of true *TBC1D3* mappings (Supplemental Fig. S5). We repeated the test by limiting our samplings to SD sites on Chromosome 17. Even with this restriction, the observed distance to *TBC1D3* resided in the bottom 3% of the simulated distribution (Fig. 3C), suggesting a nonrandom association of *TBC1D3* SDs with large chromosomal rearrangements during primate evolution.

To assess the origin of *TBC1D3* gene clusters, we sequenced and assembled the genome of an outgroup primate species using HiFi data generated from a mouse lemur (*Microcebus murinus*) and identified two sequence contigs (2.8 Mbp and 14 Mbp) spanning the region (Fig. 3D). Both clusters 1 and 2 appeared to be absent; however, the corresponding regions demarcate breakpoints of synteny compared with Old World monkey and ape lineages. Additionally, we aligned *TBC1D3* against the entire mouse lemur assembly with BLASTN but could not identify any *TBC1D3* orthologs, suggesting *TBC1D3* is exclusive to the simian infraorder (Supplemental Table S6; Zhang et al. 2000). We followed up this analysis and compared human and owl monkey *TBC1D3* orthologs by genomic synteny and phylogenetic approaches to identify the putative simian ancestral *TBC1D3* paralog but did not find a consistent candidate (Supplemental Fig. S6).

### *TBC1D3* transcript and open reading frame prediction

**Figure 4.**
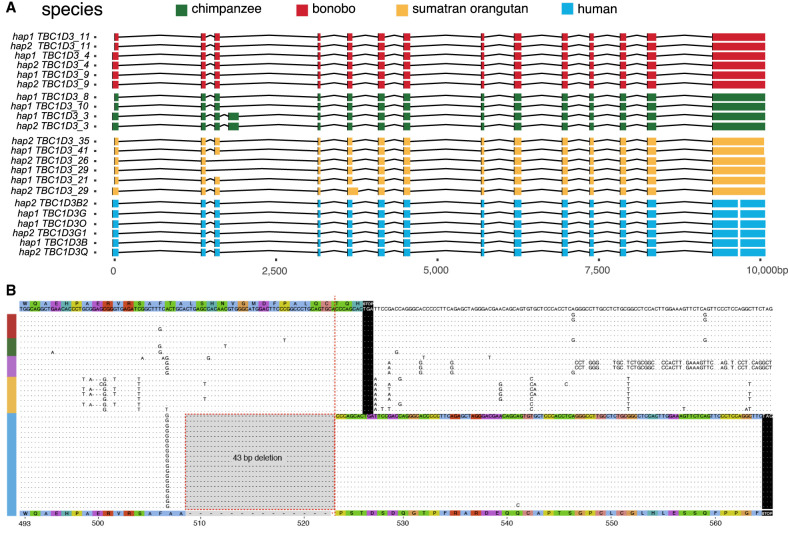
Human-specific C-terminal modification of *TBC1D3*. (*A*) The intron/exon structure of expressed *TBC1D3* isoforms with protein-encoding ORFs. Each row constitutes a paralog-specific isoform observed based on Iso-Seq (Methods). All isoforms were mapped to human *USP6* for a common reference. Exons are colored by species, with arches representing introns. (*B*) Amino acid sequence alignment of the C terminus of expressed primate *TBC1D3* paralog sequences predicted from Iso-Seq full-length cDNA. All, and only, human-expressed copies contain a 43 bp deletion within the ORF of the terminal exon, resulting in a frameshift, creating an extension of 41 novel amino acids to the C terminus.

Gene model characterization of *TBC1D3* has been particularly challenging given the high sequence identity and variable nature of the duplicated genes. This has made it difficult to distinguish genes that are expressed and potentially functional from pseudogenes. To address this limitation, we sequenced HiFi, full-length nonchimeric (FLNC) cDNA using a PacBio isoform sequencing (Iso-Seq) assay (Methods) (Dougherty et al. 2018). We generated or analyzed data from testis tissue of chimpanzees, gorillas, bonobos, and Sumatran and Bornean orangutans (Makova et al. 2024) and from pooled human fetal brain tissue (Supplemental Table S7). Additionally, we analyzed a very deep pool of about 500 million human FLNC reads recently generated from induced pluripotent stem cells (iPSCs) (Cheung et al. 2023). We mapped FLNC reads to both haplotypes of the respective species of origin genome assemblies, allowing only high-quality mappings and tracking all best map assignments versus multiple mappings among the paralogous copies for each species (Methods) (Fig. 4A). Although unambiguous one-to-one assignments between transcripts and specific paralogs could not always be made, the analysis revealed three important features. First, *TBC1D3* is transcribed in all ape lineages with evidence of multiple paralogs expressed where there are duplications (Supplemental Fig. S7). Second, the canonical 14-exon gene model is retained across the apes, with evidence of exon exaptation and exon loss for a minority subset of transcripts in chimpanzees and Sumatran orangutans (Fig. 4A). Third, the predicted open reading frame (ORF) is, in general, maintained. In humans, however, both transcription and ORF maintenance are most likely to be retained among *TBC1D3* copies mapping to clusters 1 and 2 in contrast to distal orphan copies (see Fig. 2A, human Chromosome 17 ideogram).

During our comparison of human and NHP *TBC1D3* gene models, we noted that all human transcripts harbor a 43 bp deletion in the ORF absent in NHPs (Fig. 4B). This deletion removes the last 17 amino acid residues common to NHPs and introduces a frameshift, resulting in a 41 amino acid extension and a novel C terminus of the human TBC1D3 protein. All other NHPs lack this carboxy extension owing to a shared common stop codon. We also confirmed this human-specific difference at the level of the assembly using ProSplign (Methods) (https://www.ncbi.nlm.nih.gov/sutils/static/prosplign/prosplign.html). Furthermore, the 43 bp deletion is restricted to *TBC1D3* copies mapping to human clusters 1 and 2, in which 95% (850/896) of cluster 1 and 2 copies contain the deletion, and it is not observed among the older orphan paralogs distributed throughout human Chromosome 17 (Supplemental Fig. S8). These findings indicate that this fundamental change in the ORF is human-specific and occurred during human *TBC1D3* expansion within clusters 1 and 2. We predicted the effect of this modification on the tertiary structure of TBC1D3 using AlphaFold2 but found that the novel C-terminal sequence was disordered (Supplemental Fig. S9; Jumper et al. 2021).

### African ape positive selection

**Figure 5.**
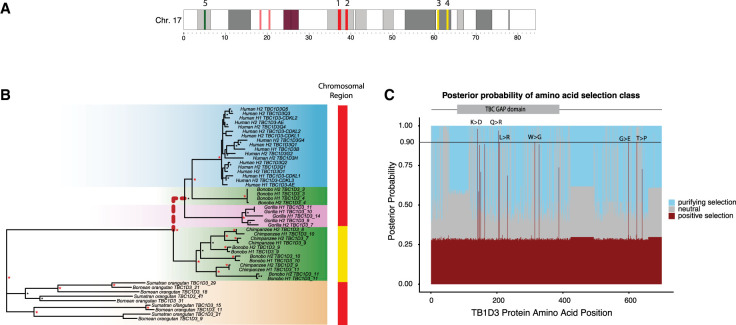
Positive selection of the *TBC1D3* gene family. (*A*) Chromosome 17 ideogram marking *TBC1D3* expansion clusters (red) and distal loci (yellow) expressed in chimpanzee and bonobo. (*B*) Branch site test of selection for expressed *TBC1D3* paralogs. A maximum likelihood phylogeny corresponding to the introns of expressed *TBC1D3* paralogs used to visualize relation of expressed copies. The red dashed branch illustrates the ancestral branch identified under positive selection with absREL (*P*-value = 0.01). Colored bars on the *right* of the phylogeny illustrate the location of origin of each *TBC1D3* copy as illustrated in *A*, red indicating paralogs from clusters 1 and 2 and yellow marking expressed paralogs from distal q-arm expansions 3 and 4. (*C*) Sites under selection along *TBC1D3*. A branch site model was conducted using the codon alignment of the same *TBC1D3* expressed isoforms, with the branch leading to African ape cluster 1 and cluster 2 *TBC1D3* copies as the foreground and all other branches as the background. Posterior probabilities for positive, neutral, and purifying selection are illustrated in red, gray, and blue, respectively, with red indicating sites under selection in the foreground branches (omega = 52.6). Six sites were observed with strong evidence of positive selection (141K > D; 205Q > R; 208L > R; 315W > G; 598G > E; 624T > P).

Using the full-length transcript isoforms that were generated and mapped to the complete genome assemblies from each primate (Fig. 5A), we constructed two MSAs using intronic sequence and codon-aligned exonic regions. First, we explored branches putatively under positive selection using a free-ratios model (Methods) (Yang 2007). We identified three branches and tested these for a significant excess of amino acid replacements using the codon MSA in an adaptive branch-site random effects likelihood test (absREL; Methods) (Supplemental Table S8; Smith et al. 2015). After multiple test correction, we found strong statistical support for positive selection in one of the three branches, within the ancestral branch leading to African ape cluster 1 and cluster 2 *TBC1D3* copies (*P* = 0.01; Methods) (Fig. 5B). This positive selection is detected only for *TBC1D3* copies mapping to clusters 1 and 2 and not among orphan copies or other ape clusters distributed along Chromosome 17. Furthermore, this selection occurred after divergence from orangutans and after an African ape–specific translocation of *TBC1D3* paralogs to Chromosome 17q23 (Fig. 3A). Orangutan copies expressed from clusters 1 and 2 do not show signatures of positive selection, nor do expressed chimpanzee/bonobo copies mapping distally to clusters 3 and 4 (yellow). Focusing on African ape copies mapping to clusters 1 and 2, we tested for site-specific signatures of positive selection on amino acid residues with a branch-site model (Methods) (Yang 2007). Using a Bayesian posterior probability cutoff of 0.9, we identified six sites of positive selection, with the strongest signals mapping within the TBC/Rab GTPase-activating protein (GAP) domain, as well as two residues proximal to the C terminus of TBC1D3 (Fig. 5C). These signals of positive selection cannot be explained by gene conversion (Supplemental Fig. S10).

### Pangenomic characterization and transcription of human *TBC1D3* copies

**Figure 6.**
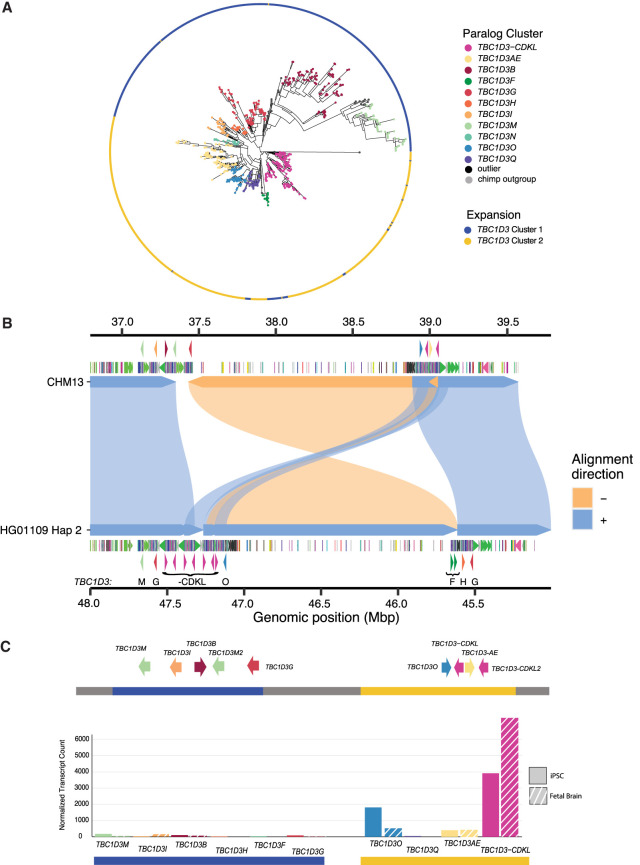
Pangenomic characterization and expression of *TBC1D3* in humans. (*A*) Maximum likelihood phylogeny of all validated *TBC1D3* cluster 1 and 2 paralogs in humans, outgrouped to chimpanzee *TBC1D3*. Individual cluster paralogs were identified by limiting intra-cluster variation to a 1.5× allelic variation observed in SD sequence. This resulted in a gene family of 11 common paralogs. (*B*) Inversion haplotype of HG01109 hap2 (*bottom*) aligned to CHM13 (*top*). (*C*) Visual illustration of CHM13 clusters 1 and 2 with new paralog characterization, as well as expression of these paralogs across iPSCs and fetal brain Iso-Seq libraries, normalized to median haplotype paralog copy number.

Given the extraordinary copy-number variation among human copies mapping to clusters 1 and 2, we applied a pangenomic approach to organize and characterize human paralogs. We initially constructed pangenome graphs with Minigraph from the sequence-resolved human haplotypes. However, few paralogs were grouped as common or shared but, instead, the majority of *TBC1D3* copies were represented as isolated nodes with single-haplotype support (Supplemental Fig. S11; Li et al. 2020). As a result, we applied a phylogenetic approach that organized *TBC1D3* copies into groups in which genetic distance exceeded the expected level of intra-allelic variation (Methods). We defined 11 distinct phylogenetic groups (Fig. 6A) and named them based on *TBC1D3* paralogs already present in the human reference genome (GRCh38) (Supplemental Fig. S12). In some cases, multiple distinct paralogs were placed into the same phylogenetic group if paralogous variation was less than the expected extent of allelic variation (e.g., *TBC1D3-AE* or *TBC1D3-CDKL*). We identified four novel phylogenetic groups representing paralogous copies not present in the human reference genome assembly: *TBC1D3M*, *TBC1D3N*, *TBC1D3O*, and *TBC1D3Q.* Most phylogenetic groups are distributed across human continental population groups and are specific to either cluster 1 or 2. *TBC1D3F*, however, is exclusive to Amerindians and maps to cluster 2, yet has greater homology with cluster 1 *TBC1D3* members. A detailed examination of the genomic organization of one of these Amerindian haplotypes, HG01109 H2, reveals that the entire 1.35 Mbp region bracketed by clusters 1 and 2 has been inverted, suggesting that inversion, as well as gene conversion, may be playing a role in relocating *TBC1D3* paralogs between clusters 1 and 2 (Fig. 6B).

Using this phylogenetic group classification of cluster 1 and 2 members, we revisited expression of the *TBC1D3* gene family in humans, taking advantage of the deep Iso-Seq data sets that had been generated from both iPSCs and fetal brain (Supplemental Table S7). We mapped FLNC reads from both sources to the phylogenetic pangenome groups and identified the best primary paralog mapping for each read (Methods). We find that the majority of *TBC1D3* expression—91% in iPSCs and 96% in fetal brain—originates from cluster 2–specific paralogs. Furthermore, the majority of this sequence—89% in fetal brain and 69% in iPSCs—maps to a single phylogenetic group: *TBC1D3-CDKL*. This enriched paralog expression is consistent, even when normalized by median *TBC1D3* paralog copy (Fig. 6C). It is noteworthy that for 67 of the 69 assembled haplotypes, this expressed *TBC1D3* paralog is the last copy in cluster 2 and, furthermore, is oriented such that the unique sequence flanking this telomeric end of the cluster is directly upstream to its transcription start site. A genome-wide analysis identified that the 20 kbp of this unique sequence falls within the lower 5% for pairwise nucleotide diversity and may reflect either a selective sweep or regulatory sequence under strong purifying selection (Supplemental Table S9). This paralog expression exclusivity may explain why a gene family predicted to be critical to cortical expansion may be so variable in copy number and structure among humans.

## Discussion

Long-read sequencing and advances in de novo genome assembly have enabled comprehensive characterization of complex, duplicated loci (Liao et al. 2023). Here, we investigated the evolution and transcription of *TBC1D3*, a “hominoid-specific” gene family functionally implicated in the proliferation of neuronal progenitors and cortical expansion and folding of the human brain (Paulding et al. 2003; Sudmant et al. 2010; Ju et al. 2016; Hou et al. 2021). Using Hifiasm and Verkko, we successfully assembled and validated 69 human haplotypes from three references (GRCh38, CHM1, T2T-CHM13) and 33 human samples across *TBC1D3* clusters 1 and 2 (Cheng et al. 2021; Rautiainen et al. 2023). We find that the human *TBC1D3* gene family is among the most copy-number-variable gene families, with >60% of human haplotypes containing a unique structural configuration at each cluster with an overall structural heterozygosity estimated at 90%. The *TBC1D3* copy number at each cluster ranges from one to 14, which we phylogenetically reduced into 11 common *TBC1D3* paralog groups—four of which were novel and not represented in either the GRCh38 or T2T-CHM13 human references (Fig. 6A; Supplemental Fig. S12).

At first glance, this incredible genetic variation of *TBC1D3* conflicts with the proposed critical function in brain cortical expansion. Leveraging a deep long-read Iso-Seq data set from two developmental contexts (iPSCs and fetal brain), we distinguished paralog expression and found that *TBC1D3* paralogs mapping to cluster 2, most notably *TBC1D3-CDKL*, account for ∼90% of assigned transcripts. We hypothesize that this restricted pattern of expression may explain how such high copy-number variation is tolerated, because only one or two copies, located at the telomeric end of *TBC1D3* cluster 2, are exclusively expressed. This model of regulation is reminiscent of the green opsin gene family on Chromosome X, in which a single locus control region promotes expression of the most proximal green opsin paralog and downstream duplicates are transcriptionally silent (Hayashi et al. 1999). In this model, many of the other *TBC1D3* paralogs are either inactive pseudogenes or “genes-in-waiting” with the potential to become the primary gene if their position within the cluster changes. Future studies investigating *TBC1D3* regulation and expression, with methods such as Fiber-seq as well as matched RNA-seq and WGS samples to correlate copy number and expression, will help elucidate the regulatory landscape of the *TBC1D3* gene family (Stergachis et al. 2020).

*TBC1D3* is just one example of approximately two dozen core duplicons, originally defined as focal points of sequence overrepresented in SD repeat graphs (Jiang et al. 2007; Marques-Bonet and Eichler 2009; Dennis et al. 2017). Several core duplicons have been associated with recurrent and independent duplications in primates, chromosomal rearrangements among apes, large-scale inversion polymorphisms in humans, and developmental disorders (Johnson et al. 2006; Zody et al. 2006a,b; Antonacci et al. 2010; Mohajeri et al. 2016; Nuttle et al. 2016; Maggiolini et al. 2019; Porubsky et al. 2022; Mao et al. 2024). *TBC1D3* is no exception. First, we found evidence of five separate lineage-specific expansions in the different primate lineages and observed that *TBC1D3* expanded specifically in humans ∼2.5 mya when the genus *Homo* transitioned from *Australopithecus*, coinciding with the onset of frontal cortical expansions in *Homo habilis* (Spoor et al. 2015). We found a 2.2 Mbp inversion between *TBC1D3* clusters in one Amerindian haplotype, consistent with ongoing nonallelic homologous recombination between inverted *TBC1D3* gene clusters, which may provide a substrate for the recurrent 17q12 microdeletion syndrome associated with renal cyst and diabetes syndrome (RCAD) (Mefford et al. 2007). Finally, we found a suite of changes in the TBC1D3 protein sequence, including positively selected amino acid changes among African apes and a significantly transformed C terminus exclusive to humans. Unlike other African apes, all human *TBC1D3* copies that we have detected as expressed harbor this modified C terminus, suggesting it may have been a key event underlying the potential neofunctionalization of the gene family in our lineage.

Functional investigations have suggested different biochemical roles for the TBC1D3 protein at the cellular level, all of which increase cell proliferation. Two functions occur in the cytosol, where TBC1D3 antagonizes ubiquitination and degradation of EGFR and IRS1 receptors, driving cell proliferation in cell culture (Wainszelbaum et al. 2008; 2012). The third, in contrast, proposes that TBC1D3 is shuttled to the nucleus in neuron progenitor cells, where it antagonizes EHMT2 methyltransferase and, as a result, epigenetically inhibits neural progenitor differentiation (Hou et al. 2021). Our work suggests that the extensive expansion of this gene family in humans has had limited dosage effect owing to the preferential expression/regulation of the distal cluster 2 copy. Instead, we propose that the human-specific modified C terminus plays a critical role in these adaptive functions by potentially directing novel post-translational modifications or altering the localization and trafficking of TBC1D3 proteins (Sharma and Schiller 2019). It will be important to compare the structure and function of human and NHP TBC1D3 proteins to determine if neofunctionalization has indeed occurred as a result of these changes in the human lineage. The power of long-read sequencing to resolve structural variation, expression, and regulation of complex gene families such as *TBC1D3* makes these fundamental questions addressable.

## Methods

### Long-read sequence and assembly

The majority of genomes used in this study were sequenced previously as part of other assembly efforts to generate phased genomes or T2T genomes and are publicly available under NCBI BioProject (https://www.ncbi.nlm.nih.gov/bioproject/) accession numbers PRJNA941350, PRJNA877605, PRJNA941358, PRJNA916732, PRJNA916733, PRJNA916735, PRJNA916734, PRJNA916736, and PRJNA916737 (Liao et al. 2023; Makova et al. 2024; Mao et al. 2024). For species, coverage, and project details, see Supplemental Table S10. This study focused only on analyzing sequence contigs that contained copies of *TBC1D3* paralogs, and we evaluated each contig for gaps and contiguity (see Assembly validation section below). Most human genomes were originally assembled using Hifiasm (version 0.15.2), but *TBC1D3-*containing contigs that failed QC were reassembled with Verkko (versions 1.0, 1.1, 1.2, and 1.4) using a combination of both HiFi and ONT sequence. In general, haplotypes were phased using parental *k*-mer information when available, or Hi-C chromatin capture data (Auton et al. 2015; Kronenberg et al. 2021). For the Chromosome 17 comparison, it was observed that the macaque orthologous chromosome was fragmented and was subsequently scaffolded using RagTag (version 2.1.0) with the Mmul10 reference as the scaffold (Hughes et al. 2012; Alonge et al. 2022). In this study, we generated assemblies for only two species: gelada (*Theropithecus gelada*) and mouse lemur (*M. murinus*). High-molecular-weight DNA was prepared from peripheral blood of a male gelada (*DRT_2020_14_TGE*) and from skin fibroblasts of a female mouse lemur (*Inina_MMUR*). HiFi sequence data (50×, 30×) were generated using the Sequel II platform, and assemblies were generated with Hifiasm (Supplemental Table S10).

### Assembly validation

#### Illumina copy-number validation

Sample assemblies were first validated using diploid assembly *TBC1D3* copy-number estimates to Illumina sequence copy-number estimates, an orthogonal sequencing approach (Supplemental Fig. S2). Sample genome haplotypes were merged and *k*-merized into 32 bp *k*-mers using Meryl (version 1.3) (Rhie et al. 2020). In parallel, sample Illumina sequence libraries were similarly *k*-merized into 32 bp with Meryl. Next, *k*-mer libraries were aligned to the T2T-CHM13 reference genome using FastCN, allowing for up to two mismatches between the *k*-mer and assembly alignments (Pendleton et al. 2018; Nurk et al. 2022). We estimated the copy number of *TBC1D3* by taking the average copy number over one *TBC1D3* paralog, *TBC1D3L*, and compared these estimates against one another in a scatter plot (Fig. 1A; Supplemental Fig. S2).

#### Self-read mapping validation

We also applied NucFreq (Vollger et al. 2019) to assess the integrity of each *TBC1D3* assembly. Each sample's respective HiFi sequencing libraries were trio phased using Canu (version 2.1.1) (Koren et al. 2017) and mapped back onto their respective de novo assemblies. To qualitatively validate assembly, we plotted the sequence depth of both the primary and secondary bases of reads aligned over the *TBC1D3* expansions (Supplemental Fig. S1). First, we removed samples with obvious gaps over the *TBC1D3* expansion 1 and 2 loci, which could be identified if the locus was broken across multiple contigs or if the assemblies had a lack of HiFi sequence support over a given region. Next, we identified assemblies with collapses over the *TBC1D3* expansion 1 and 2 regions by looking at secondary base read depth. HiFi sequencing is 99.9% accurate, with occasional low-frequency false base calls. Our expectation is that this frequency can be observed over a given region as the secondary base, remaining well below 1% frequency. Any haplotypes with a noticeable increase in secondary base frequency over particular stretches were marked as collapsed. Usually, these samples included a spike in primary base coverage as well as over the collapsed region. Additionally, Hifiasm samples were validated with GAVISUNK (Dishuck et al. 2023). Phased ONT reads were mapped over each sample's respective assemblies, and singly unique nucleotide *k*-mer anchors were marked. We expect, for correct assemblies, that every region of the assembly will be supported by at least one ONT sequence, which is not used during Hifiasm assembly. Any locations with a gap in ONT assemblies were marked as not validated.

### Repeat and gene mapping annotation

We defined repeat content in the genome using Tandom Repeat Finder (TRF) (version 4.09; Benson et al. 1999) for simple tandem repeats, RepeatMasker (version 4.1.2-p1; http://www.repeatmasker.org) for common transposon and retrotransposon elements, and DupMasker to define duplicons associated with human SDs (Jiang et al. 2008). *TBC1D3* loci were identified in the GRCh38 reference genome based on RefSeq annotations and mapped to other assemblies using minimap2 (version 2.24), using the asm20 standardized setting and allowing for up to 1000 secondary alignments (Li 2018). These mappings were filtered to contain at least 6 kbp of sequence over half the length of the canonical *TBC1D3* gene model. For more distantly related lineages, including the New World monkeys, we mapped *TBC1D3* sequence using BLAT (version 3.5), allowing a maximum intron length of 5 kbp, half the *TBC1D3* gene model length, and a minscore of 100. These relatively loose mapping constraints identified many candidate *TBC1D3* paralogs, more than expected by either Illumina- or assembly-based *TBC1D3* copy-number estimates, that were subsequently filtered based on expression, divergence, or minimum length match.

### Structural variation and heterozygosity characterization

Validated cluster 1 and 2 *TBC1D3* haplotypes were aligned to one another in an all-by-all fashion using minimap2 (version 2.24) auto settings -x asm5, allowing up to 1 kbp of insertions in cigar strings. We labeled two haplotypes as structurally equivalent if ≥90% of their sequence could be mapped to one another in a single alignment. We repeated this exercise for all pairs of haplotypes, calculated the number of valid haplotypes with no structurally equivalent pair, and divided by the total number of validated haplotypes to determine our structural variation statistic. For structural heterozygosity, we identified all samples whose two haplotypes were not structurally equivalent and divided by the total assembled samples. Contig and chromosome alignments (e.g., Figs. 3 and 5) were visualized by SVByEye using either plotMiro for pairwise alignment, or plotAVA for all-versus-all alignments (https://github.com/daewoooo/SVbyEye). Blue alignments represent directly orientated alignments, and yellow indicates inverted alignments. For local *TBC1D3* structure comparison (Supplemental Fig. S4), we extracted primate *TBC1D3* copies, along with 25 kbp of flanking sequence, from five primate lineages and mapped to one another. These copies were organized to reflect the closest alignments, by both length and identity.

### *TBC1D3* breakpoint simulation

We mapped orthologous Chromosome 17 relationships and annotated *TBC1D3* copies using minimap2 -x asm20. Synteny was annotated using Asynt get.synteny.blocks.multi command, with max_gap = 200,000, min_block_size = 1,000,000, and min_subblock_size = 50,000, producing a tab-delimited file marking the target and query breaks of blocks (Kim et al. 2022). For each *TBC1D3* copy, we identified the nearest synteny break along the respective chromosome and then computed median distance to synteny breaks of all *TBC1D3* mappings. Next, we conducted a permutation experiment. For each primate orthologous Chromosome 17, we randomly selected ∼11 kbp blocks at the same quantity as the number of *TBC1D3* mappings observed in the respective primate chromosome. We repeated the median distance experiment and plotted the distribution of 5000 permutations.

### Multiple sequence alignment

Sequence was extracted from assemblies by mapping *TBC1D3* sequence to full genome assemblies with minimap2 (version 2.24) and extracting the mapped reference sequence with BEDTools (version 2.29.2) (Quinlan and Hall 2010; Li 2018). MSAs were constructed with MAFFT with parameters ‐‐reorder ‐‐maxiterate 1000 ‐‐thread 16 (version 7.453) (Katoh et al. 2002). Following MSA construction, spurious alignments were pruned with trimmal (‐‐gappyout; version 1.4) and manually trimmed. Codon alignments were generated with matched ORF and amino acid sequence FASTA files. First, an amino acid MSA was generated with MAFFT, and then the ORF FASTA was aligned to the amino acid MSA with pal2nal (Suyama et al. 2006).

### Phylogenetic analyses

Maximum likelihood phylogenies were generated with iqtree2 using model setting -m MFP, 1000 lrt replicates, and -b 1000 replicates for bootstrap (version 2.1.2). Additionally, each phylogeny generated was outgrouped to a sequence: marmoset *TBC1D3* for primate phylogenetic analysis and chimpanzee *TBC1D3* for human paralog clustering. Phylogenetic trees were illustrated in R with ggtree (Yu 2023). Timing estimates for individual primate expansions were conducted using BEAUTi for data input and BEAST2 for computation (Drummond et al. 2012; Bouckaert et al. 2019). We used human–macaque and human–chimpanzee divergence times of 25 and 6.5 mya, estimated by the fossil record, as benchmarks for the computation (Dunsworth 2010; Stevens et al. 2013). With these references, we calculated the 95% confidence intervals of mutation rate within sequences and then estimated species-specific expansions with this mutation rate as well as branch lengths of the primate phylogeny. For tests of positive selection, we isolated intronic sequence and exonic sequence from paralog isoforms with expression support from the human, chimpanzee, bonobo, gorilla, Sumatran orangutan, and Bornean orangutan genome assemblies.

We tested for positive selection in coding sequence using both the PAML package and absREL (Yang 2007; Smith et al. 2015). We focused on *TBC1D3* paralog isoforms for which there was evidence of transcription based on Iso-Seq FLNC analysis from the human, chimpanzee, bonobo, gorilla, Sumatran orangutan, and Bornean orangutan samples. To serve as a proxy for neutral evolution, we isolated 7245 bp of intronic sequence from each expressed paralog and generated an MSA and maximum likelihood phylogeny, with orangutan *TBC1D3* copies as our outgroup. In parallel, we extracted 1884 bp of exonic sequence, predicted amino acid sequence with ORFipy, and codon-aligned exonic sequence with Pal2Nal (Suyama et al. 2006). With the intronic phylogeny and codon-aligned MSA, we identified branches undergoing accelerated evolution with a free-ratios model, in which independent *d*_N_/*d*_S_ values are computed for each branch in the tree (Yang 2007). We ignored predicted *d*_N_/*d*_S_ values for terminal branches, as too few changes occurred, and they were underpowered to detect selection. Among deeper branches, we identified three that were predicted to be under selection, as discussed in the text. We more stringently tested these three branches with the absREL test hosted on hyphy, which infers the optimal number of omega values and tests branches under positive selection with a likelihood ratio test statistic (Supplemental Table S8; https://stevenweaver.github.io/hyphy-site/methods/selection-methods/). After multiple test corrections, we identified one branch under positive selection. For site-level resolution, we isolated this branch in a branch-site model test and selected the amino acid residues under selection using the Bayes empirical Bayes posterior probability (Yang et al. 2005).

### Iso-Seq and transcript analyses

Primate Iso-Seq testis data were generated by Makova et al. (2024) and made available from the NCBI Sequence Read Archive (SRA; https://www.ncbi.nlm.nih.gov/sra) under accession numbers SRX18421140, SRX18280098, SRX18280097, SRX19199753, SRX19199753, and SRX18421141. Similarly, human iPSC Iso-Seq was previously generated by Cheung et al. (2023) and made available from the database of Genotypes and Phenotypes (dbGaP; https://www.ncbi.nlm.nih.gov/gap/) under accession number phs002206.v4.p1. Fetal brain tissue was derived from 59 spontaneously aborted fetuses with sequence available from SRA under accesion number SRR28199631. This sequence was enriched for both *TBC1D3* and *NPIPA1*, using the hybridization capture protocol described by Dougherty et al. (2018), with probes provided in Supplemental Table S11. FLNC libraries were mapped to respective species libraries with minimap2 using the parameters -ax splice ‐‐sam-hit-only ‐‐secondary = yes -p 0.5 ‐‐eqx -K 2G -G 8k -N 20. FLNC libraries were first filtered for reads ≥1000 bp in length and with sequence quality of ≥99.9%. Each library was subsequently mapped to the genome assembly corresponding to the respective species of origin using SAMtools (Danecek et al. 2021) and BEDTools. Next, we determined which *TBC1D3* paralogs were likely expressed by selecting paralogs with read support with mapping quality ≥99.9% sequence identify. These reads were subsequently reduced into common isoforms with IsoSeq3 (4.0.0, PacBio; https://github.com/ylipacbio/IsoSeq3) collapse, and ORFs were predicted with Orfipy (Singh and Wurtele 2021). For primate *TBC1D3* gene model comparison, isoforms with at least three independent reads of support and with the longest maintained ORF were compared. We required these reading frames to span within 100 bp of the canonical *TBC1D3* start and stop as defined by RefSeq (O'Leary et al. 2016). Human FLNC reads from fetal brain and iPSCs were mapped to all validated human haplotypes. Next, we compared these primary alignments to one another and considered the cluster paralog from which they were derived. Any Iso-Seq read with primary minimap2 alignment scores of 10 or greater for a given paralog cluster relative to all other cluster mappings was retained, whereas other mappings were marked as ambiguous and ignored.

### Analysis of coding sequence

To validate the observed deletion of coding sequence in humans, we selected human TBC1D3L amino acid sequence and mapped this sequence to all genome assemblies with ProSplign (https://www.ncbi.nlm.nih.gov/sutils/static/prosplign/prosplign.html), a tool that predicts DNA sequence representing the codons for a given protein amino acid sequence. ProSplign predicts splice junctions, as well as start and stop codons, and illustrates amino acid substitutions, frameshift mutations, and deletions in the underlying nucleotide sequence that are inconsistent with the provided amino acid sequence. We predicted the human *TBC1D3* tertiary structure using the EMBL-EBI AlphaFold2 database (Jumper et al. 2021; https://alphafold.ebi.ac.uk/). The predicted tertiary structure was illustrated using PyMOL (2.0, https://www.pymol.org).

### Human pangenome graph construction

We built a pangenome graph of *TBC1D3* with Minigraph (version 0.20; Li et al. 2020), with the settings -S -xggs -L 250 -r 100000 -t 16. We attempted graph construction with lower -l and -g settings as well but consistently observed that most haplotype *TBC1D3* paralogs were isolated to nodes without any allelic overlap from other human haplotypes.

### Human *TBC1D3* paralog grouping

We generated a phylogeny with the whole *TBC1D3* sequence for all cluster 1 and 2 copies identified in validated human assemblies, outgrouped to chimpanzee *TBC1D3*. We defined a heuristic cutoff based on allelic variation to define our clusters. Vollger et al. (2023) previously predicted allelic variation of 15.3 single-nucleotide variants per 10 kbp. We recursively identified clades with an intra-variation of up to 1.5 times the allelic variation identified in SDs. Additionally, we required that a given cluster have at least 10 independent paralogs of representation to be defined as a population-level paralog group.

## Data access

Gelada sequence and assembly data generated in this study have been submitted to the NCBI BioProject database (https://www.ncbi.nlm.nih.gov/bioproject/) under accession numbers PRJNA1081468 and PRJNA1081469. Mouse lemur sequence and assembly data generated in this study have been submitted to the NCBI BioProject database under accession numbers PRJNA1082315 and PRJNA1082316. Assembled contigs corresponding to the *TBC1D3* genomic regions for both the gelada and mouse lemur are also available at Zenodo (https://doi.org/10.5281/zenodo.12808906). Gelada and mouse lemur sequencing data used for these assemblies have been submitted to the NCBI Sequence Read Archive (SRA; https://www.ncbi.nlm.nih.gov/sra) under accession numbers SRR28199625–SRR28199630 and SRR28217961–SRR28217966, respectively. Fetal brain Iso-Seq data generated in this study have been submitted to the BioProject database under accession number PRJNA659539 and are available from the NCBI Sequence Read Archive (SRA; https://www.ncbi.nlm.nih.gov/sra) under accession number SRR28199631.

## Supplemental Material

Supplement 1

Supplement 2

Supplement 3

Supplement 4

Supplement 5

Supplement 6

Supplement 7

Supplement 8

Supplement 9

Supplement 10

Supplement 11

Supplement 12

Supplement 13

Supplement 14

Supplement 15

Supplement 16

Supplement 17

Supplement 18

Supplement 19

Supplement 20

Supplement 21

Supplement 22

Supplement 23
