# Supplementary material for: Independent expansion, selection, and hypervariability of the TBC1D3 gene family in humans
Source: Genome Res. 2024 Nov;34(11):1798–810. doi: 10.1101/gr.279299.124 (PMC11610581; doi:10.1101/gr.279299.124)
Supplement: Supplement 1 [file Supplemental_Fig_S1.pdf]

A

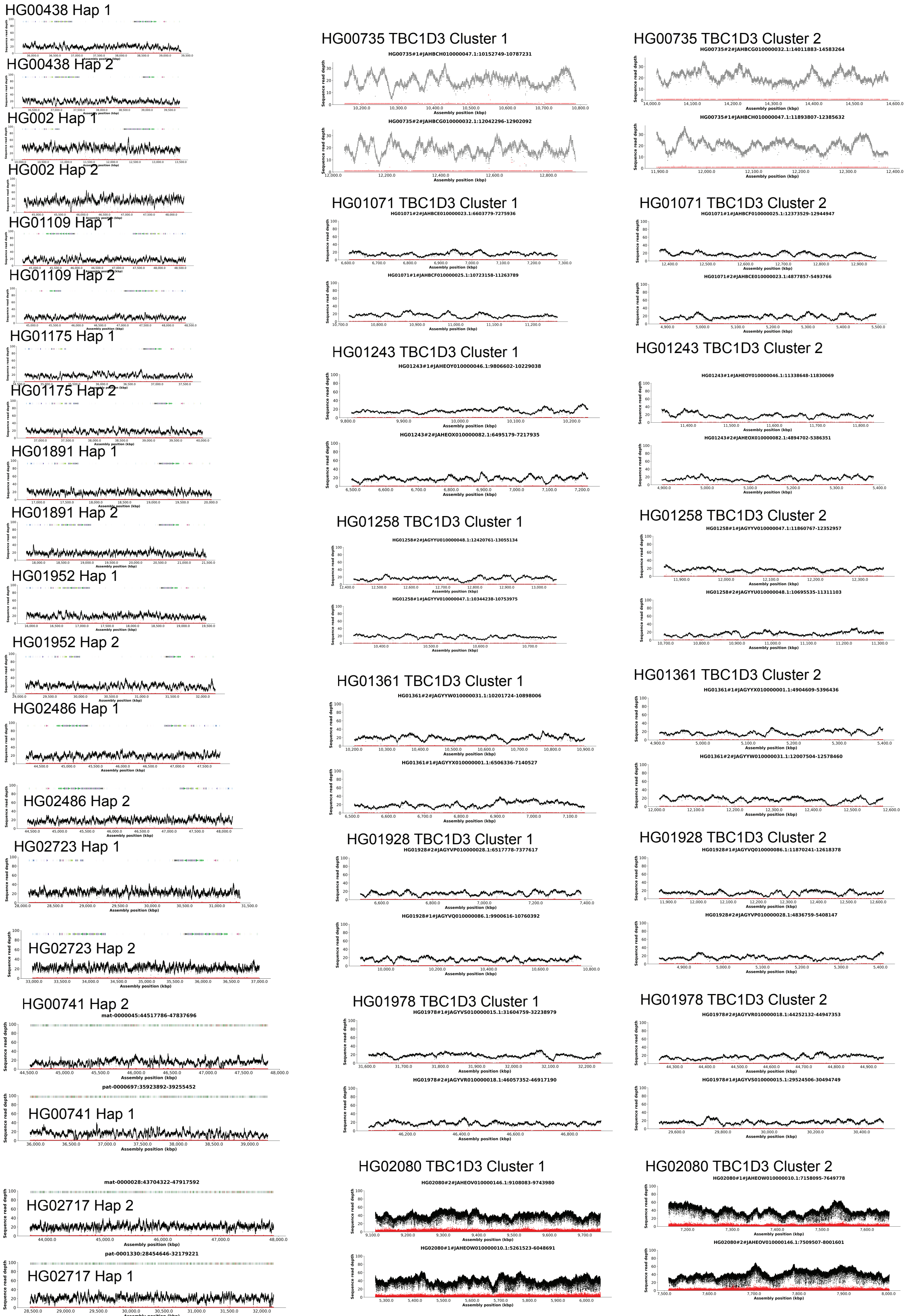
HG00438 Hap 1
HG00438 Hap 2
HG002 Hap 1
HG002 Hap 2
HG01109 Hap 1
HG01109 Hap 2
HG01175 Hap 1
HG01175 Hap 2
HG01891 Hap 1
HG01891 Hap 2
HG01952 Hap 1
HG01952 Hap 2
HG02486 Hap 1
HG02486 Hap 2
HG02723 Hap 1
HG02723 Hap 2
HG00741 Hap 2
HG00741 Hap 1
HG02717 Hap 2
HG02717 Hap 1
HG00735 TBC1D3 Cluster 1
HG00735 TBC1D3 Cluster 2
HG01071 TBC1D3 Cluster 1
HG01071 TBC1D3 Cluster 2
HG01243 TBC1D3 Cluster 1
HG01243 TBC1D3 Cluster 2
HG01258 TBC1D3 Cluster 1
HG01258 TBC1D3 Cluster 2
HG01361 TBC1D3 Cluster 1
HG01361 TBC1D3 Cluster 2
HG01928 TBC1D3 Cluster 1
HG01928 TBC1D3 Cluster 2
HG01978 TBC1D3 Cluster 1
HG01978 TBC1D3 Cluster 2
HG02080 TBC1D3 Cluster 1
HG02080 TBC1D3 Cluster 2
Sequence read depth
Assembly position (kbp)

B

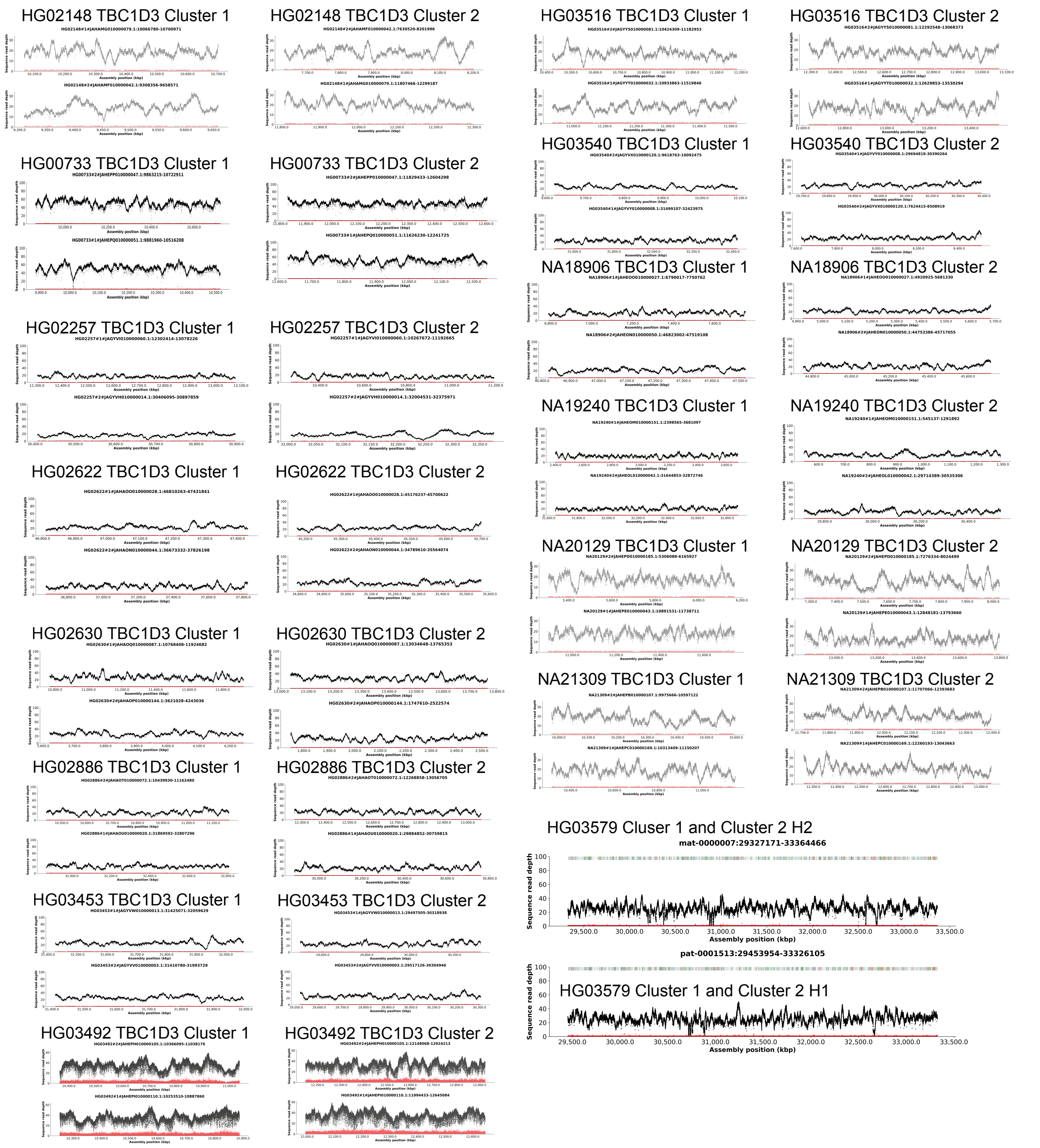

Supplemental Figure S1: NucFreq validation. Haplotype assembly accuracy was tested with NucFreq. Here, accuracy over each haplotype of *TBC1D3* cluster 1 and cluster 2 is demonstrated. Endogenous HiFi reads used to assemble respective phylogenies were mapped back onto the respective assemblies. We illustrated primary and secondary most prevalent base calls for reads aligned over a portion of an assembly in black and red, respectively. We expect primary base-call coverage to be relatively consistent, and consistently higher than the secondary base call, which should illustrate the sparse inaccuracies of HiFi sequencing.
