# Supplementary material for: Independent expansion, selection, and hypervariability of the TBC1D3 gene family in humans
Source: Genome Res. 2024 Nov;34(11):1798–810. doi: 10.1101/gr.279299.124 (PMC11610581; doi:10.1101/gr.279299.124)
Supplement: Supplement 2 [file Supplemental_Fig_S2.pdf]

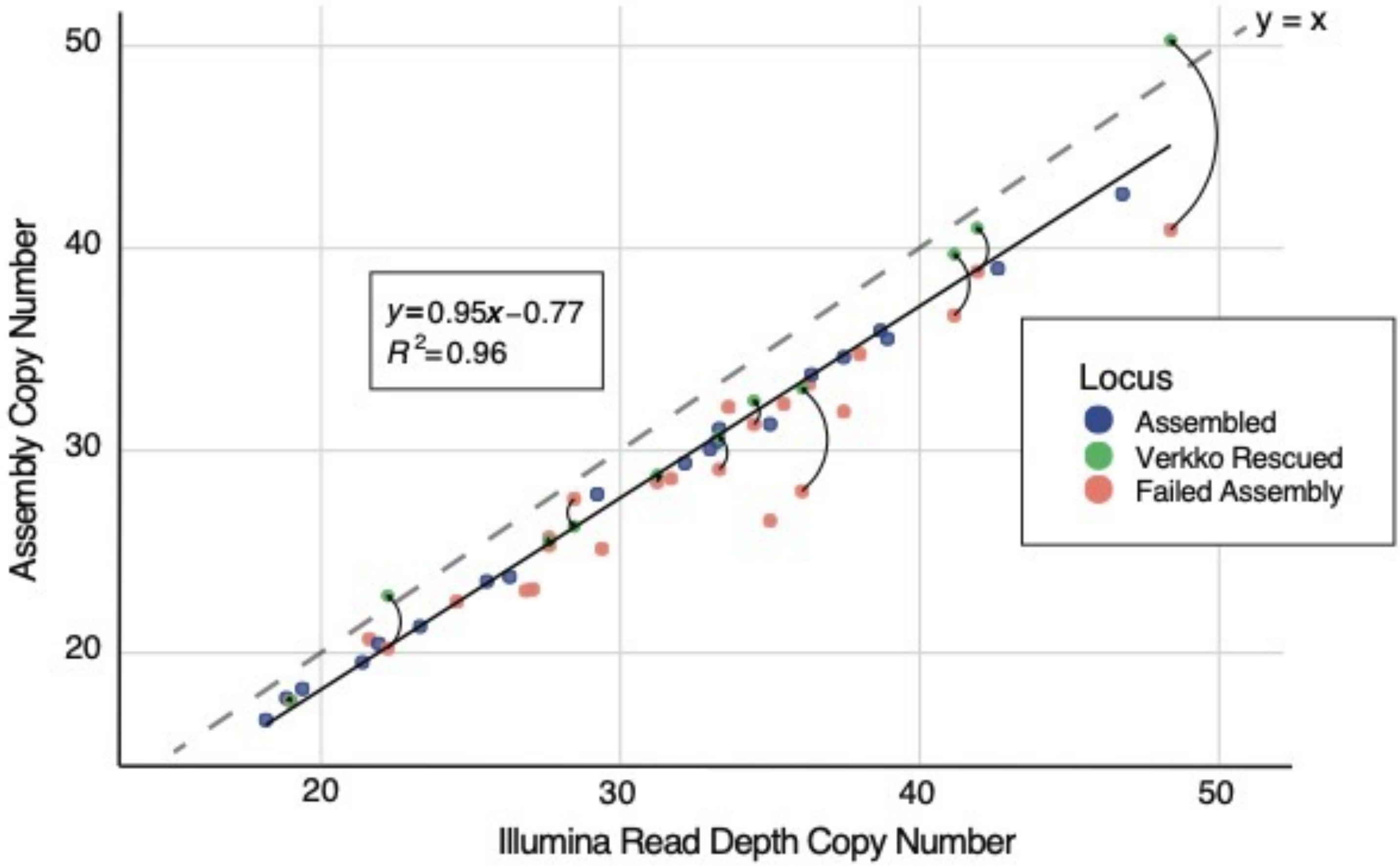

Supplemental Figure S2: Assembly and rescue of *TBC1D3* phased haplotypes. Samples were first phased and assembled using exclusively HiFi sequence. The y-axis represents the diploid copy number of these assemblies, and the x-axis represents diploid copy number based on Illumina sequence, with y=x axis marked with a dashed line. Assemblies were validated by read depth estimates of HiFi and ONT, colored in blue if validated, and salmon colored if failing validation. We attempted to rescue sample haplotypes using a novel assembly approach leveraging both HiFi and ultra-long ONT. These samples, assembled by Verkko, are indicated in green.
