# Supplementary material for: Independent expansion, selection, and hypervariability of the TBC1D3 gene family in humans
Source: Genome Res. 2024 Nov;34(11):1798–810. doi: 10.1101/gr.279299.124 (PMC11610581; doi:10.1101/gr.279299.124)
Supplement: Supplement 3 [file Supplemental_Fig_S3.pdf]

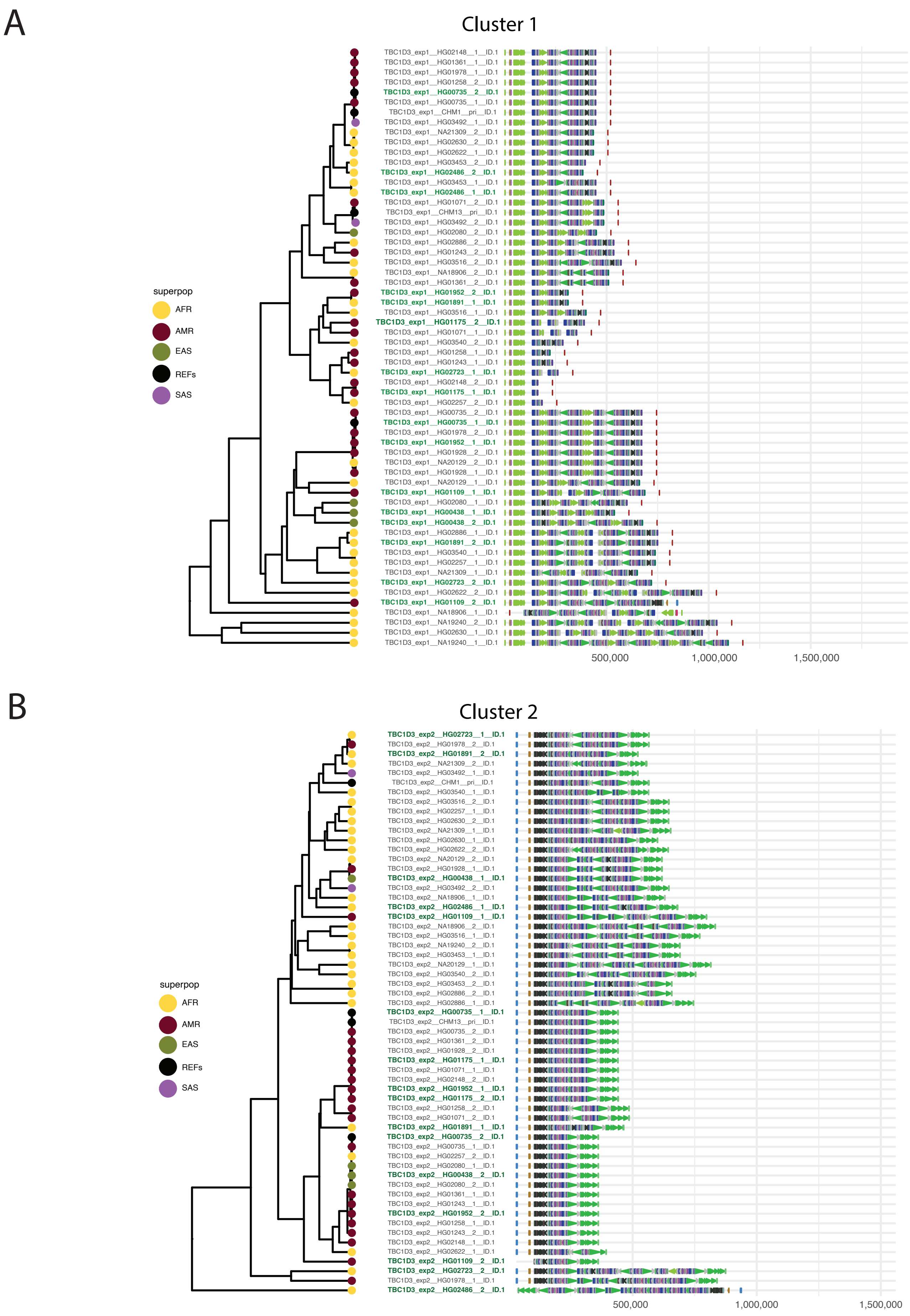

Supplemental Figure S3: UPGMA clustering of *TBC1D3*. We hierarchically clustered validated assemblies based on duplicon content using UPGMA for cluster 1 **(A)** and 2 **(B)** (Methods). Superpopulations for each haplotype are included in the dendrogram, and assemblies rescued with Verkko are colored in green.
