# Supplementary material for: Independent expansion, selection, and hypervariability of the TBC1D3 gene family in humans
Source: Genome Res. 2024 Nov;34(11):1798–810. doi: 10.1101/gr.279299.124 (PMC11610581; doi:10.1101/gr.279299.124)
Supplement: Supplement 4 [file Supplemental_Fig_S4.pdf]

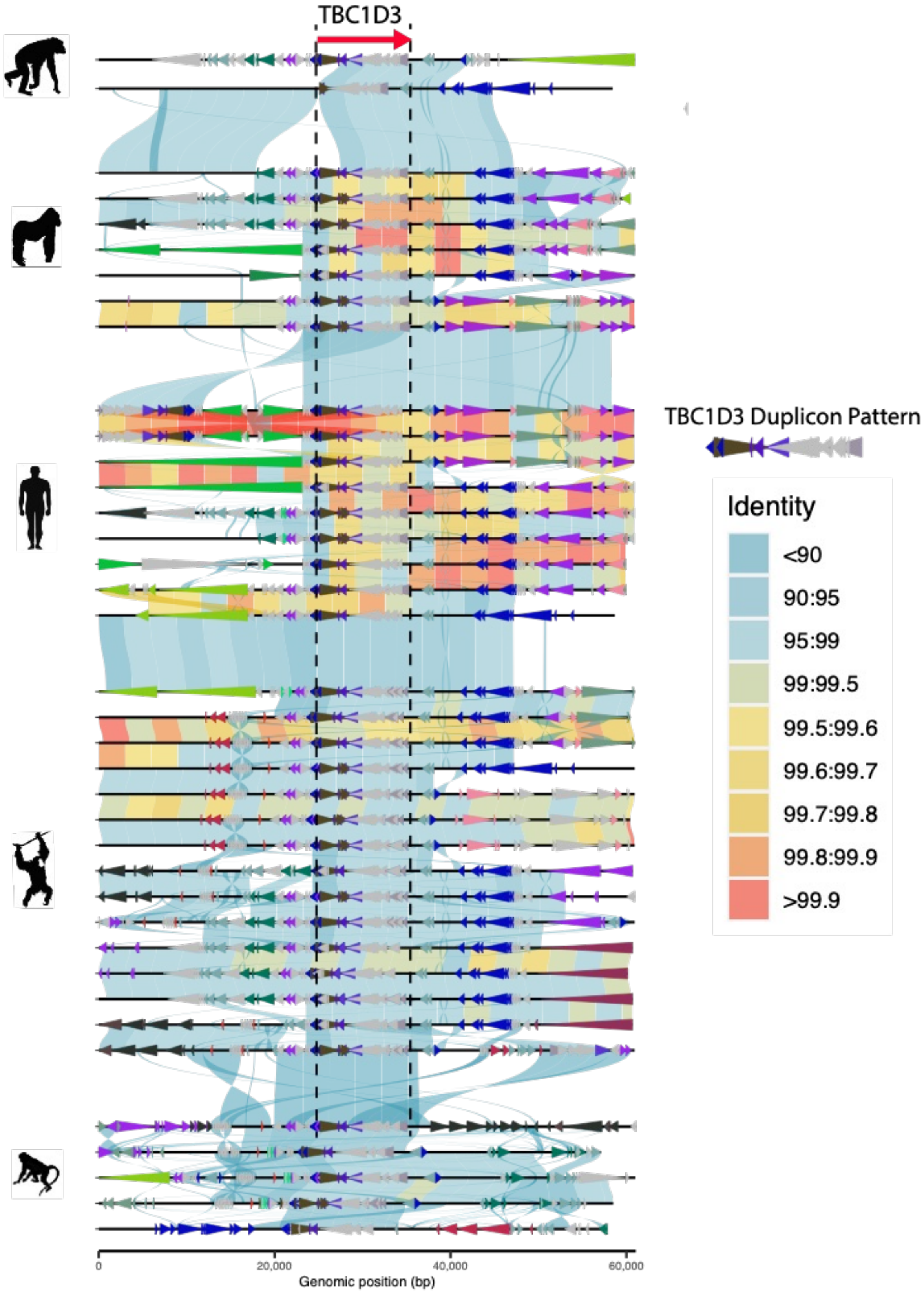

Supplemental Figure S4: Local *TBC1D3* duplicon structure supports independent expansion. *TBC1D3* copies from clusters 1 and 2, along with 25 kbp flanking sequence, were extracted and mapped to one another. We observe that these paralogs consistently map best, with highest sequence identity and contiguity, to paralogs from their same species of origin, consistent with the hypothesis of independent expansion. This contiguity is similarly observed in underlying duplicon content, shown with colored arrows and annotated with DupMasker.
