# Supplementary material for: Independent expansion, selection, and hypervariability of the TBC1D3 gene family in humans
Source: Genome Res. 2024 Nov;34(11):1798–810. doi: 10.1101/gr.279299.124 (PMC11610581; doi:10.1101/gr.279299.124)
Supplement: Supplement 5 [file Supplemental_Fig_S5.pdf]

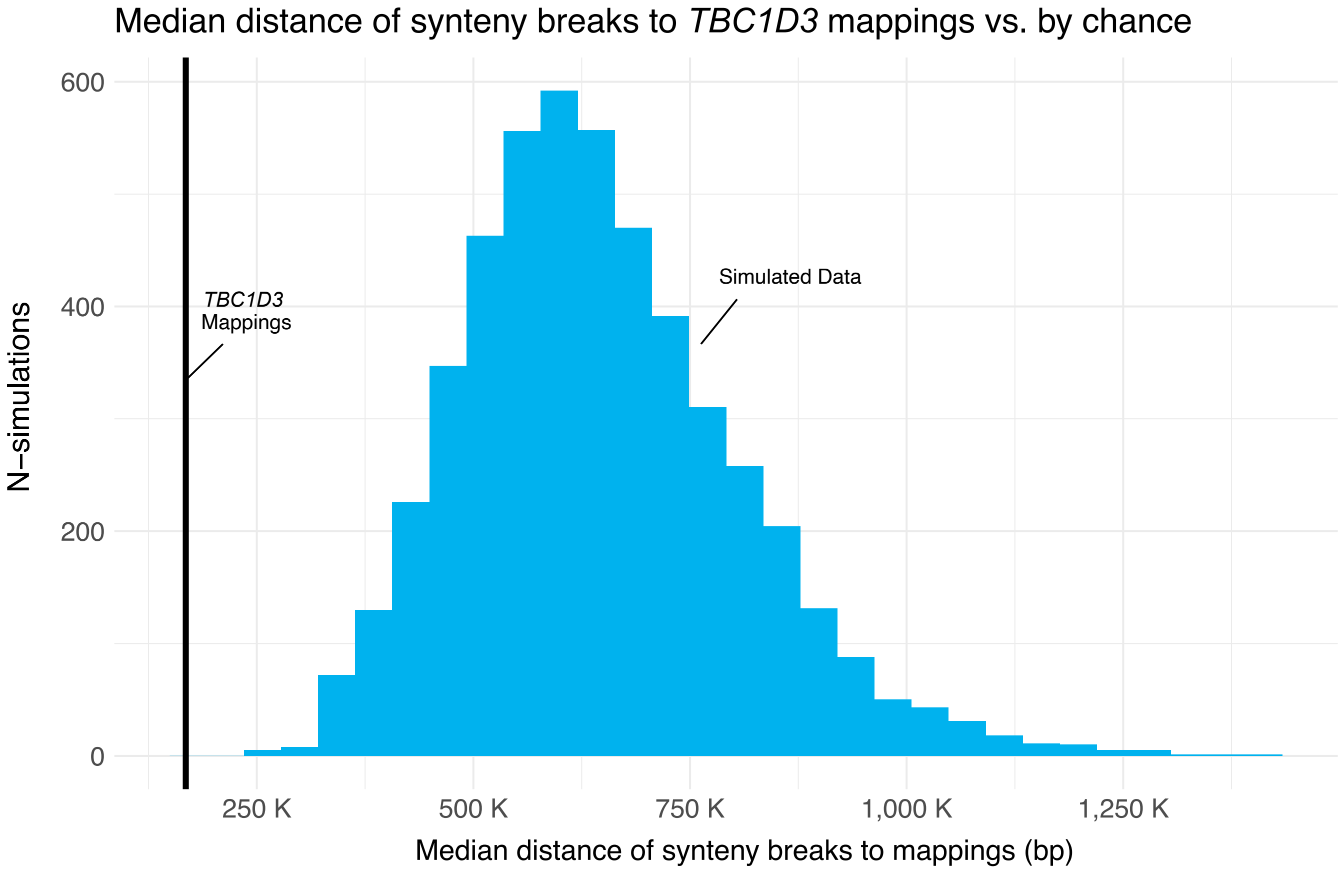

Supplemental Figure S5: *TBC1D3* vs. random genomic sequence permutation. Sequences of 11 kbp were randomly selected from orthologous primate Chromosome 17 contigs at the same quantity as the observed *TBC1D3* copies contained within the chromosome. We calculated the median distance of this sampling and repeated this experiment in 5000 permutations, comparing median distance relative to true *TBC1D3* mappings, marked in black.
