# Supplementary material for: Independent expansion, selection, and hypervariability of the TBC1D3 gene family in humans
Source: Genome Res. 2024 Nov;34(11):1798–810. doi: 10.1101/gr.279299.124 (PMC11610581; doi:10.1101/gr.279299.124)
Supplement: Supplement 6 [file Supplemental_Fig_S6.pdf]

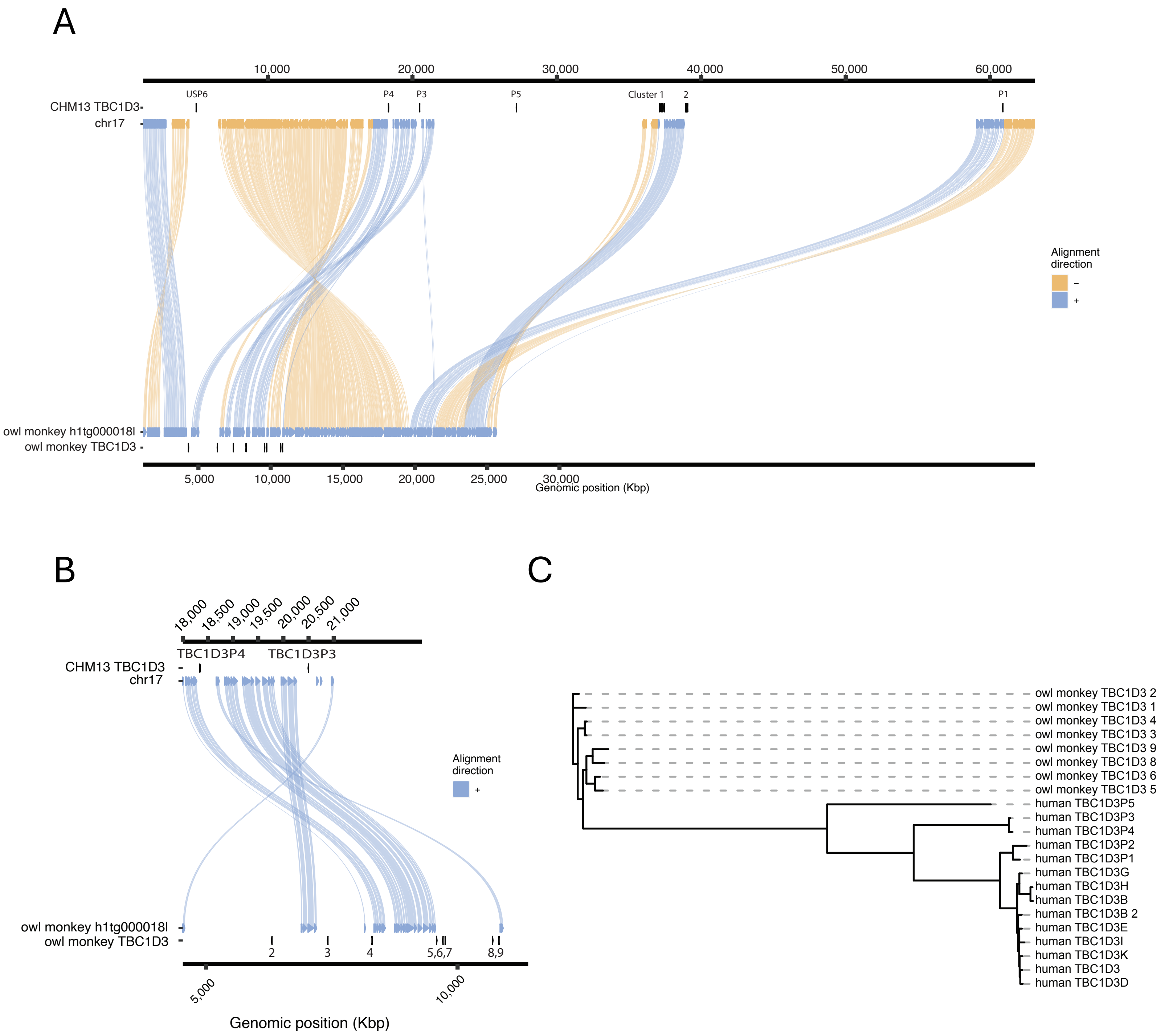

Supplemental Figure S6: Search for ancestral simian *TBC1D3* in human and owl monkey assemblies: (A) Human Chr. 17 was aligned to the single owl monkey contig marked with *TBC1D3* copies to identify synteny at *TBC1D3* locations. **(B)** Zoomed-in synteny between human *TBC1D3P4* and *TBC1D3P3* with several owl monkey paralogs suggests human *TBC1D3P3* or *P4* may represent the ancestral copy. **(C)** Maximum likelihood phylogeny of all human vs. owl monkey *TBC1D3* paralogs contradicts **(B)** suggesting *TBC1D3P5* may in fact represent the ancestral copy in humans.
