# Supplementary material for: Independent expansion, selection, and hypervariability of the TBC1D3 gene family in humans
Source: Genome Res. 2024 Nov;34(11):1798–810. doi: 10.1101/gr.279299.124 (PMC11610581; doi:10.1101/gr.279299.124)
Supplement: Supplement 7 [file Supplemental_Fig_S7.pdf]

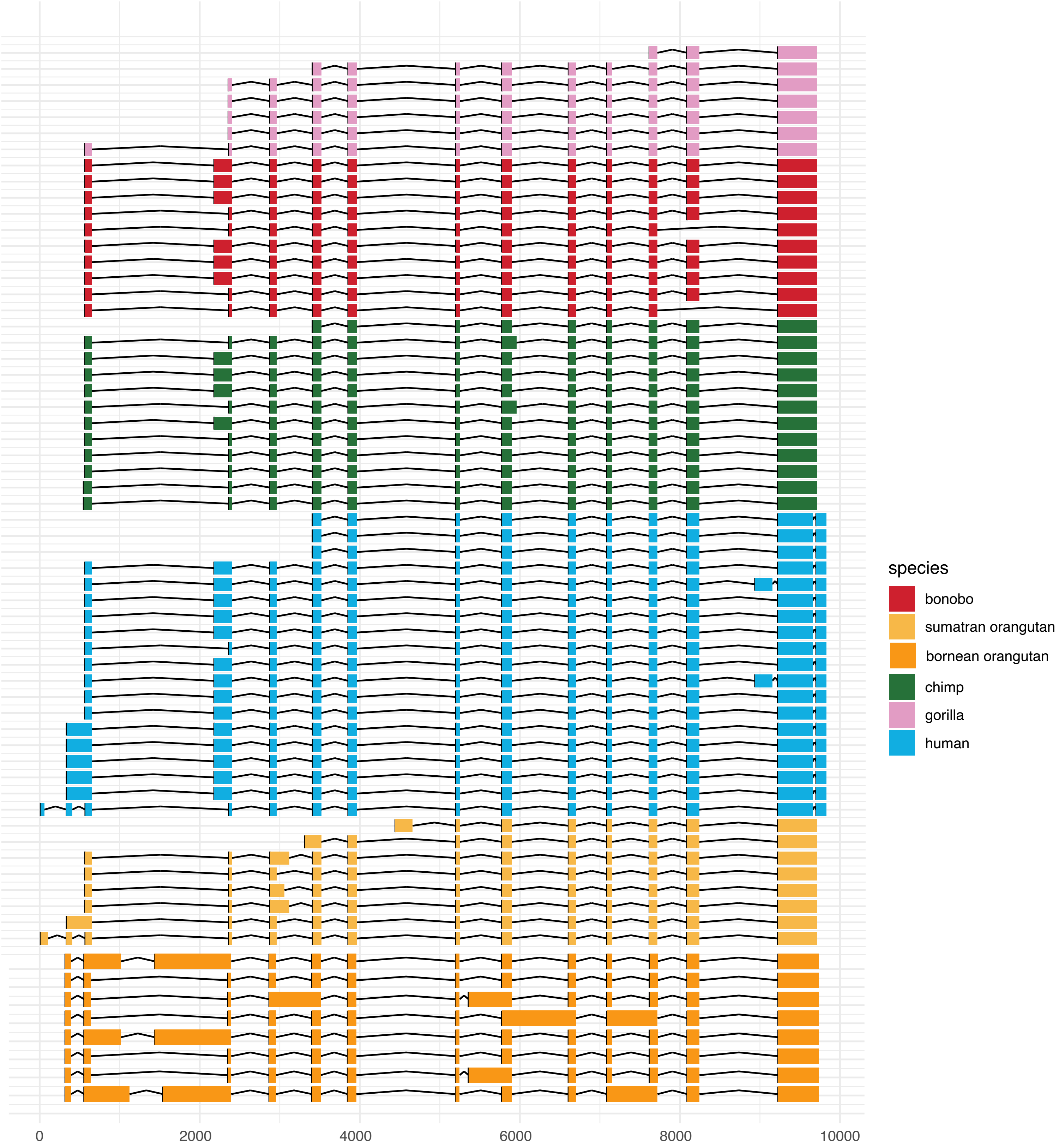

Supplemental Figure S7: Expressed *TBC1D3* paralog isoforms. Paralog-specific isoforms were selected for each primate based on their length, mapping quality, and expression support. We observe expression of *TBC1D3* from all ape lineages examined; however, for ORF analysis Bornean orangutan and gorilla isoforms were removed due to lack of expression support or intron retention.
