# Supplementary material for: Independent expansion, selection, and hypervariability of the TBC1D3 gene family in humans
Source: Genome Res. 2024 Nov;34(11):1798–810. doi: 10.1101/gr.279299.124 (PMC11610581; doi:10.1101/gr.279299.124)
Supplement: Supplement 8 [file Supplemental_Fig_S8.pdf]

T2T TBC1D3 mapping::chr17:60876851-60887769(-) TBC1D3L terminal exon amino acid sequence

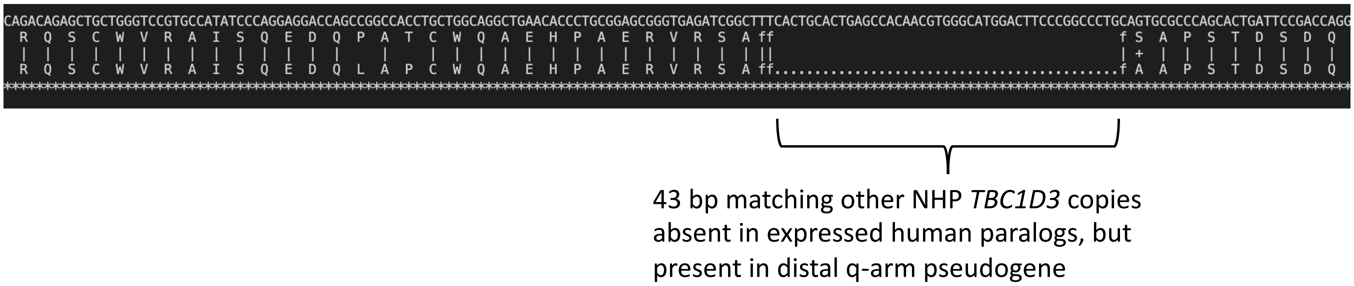

Supplemental Figure S8: Missing 43 bp deletion in human distal *TBC1D3* pseudogenes. Human-expressed *TBC1D3*, with ORF modified by a 43 bp deletion, was mapped to all primate *TBC1D3* copies, including all human *TBC1D3* copies, with prospligm (Kiriyutin et al. 2017). Prospligm predicts the genomic nucleotides that represent codons of a given amino acid. We observe that distal *TBC1D3* pseudogenes lack the 43 bp deletion universally present in cluster 1 and 2 human *TBC1D3* copies.
