# Supplementary material for: Independent expansion, selection, and hypervariability of the TBC1D3 gene family in humans
Source: Genome Res. 2024 Nov;34(11):1798–810. doi: 10.1101/gr.279299.124 (PMC11610581; doi:10.1101/gr.279299.124)
Supplement: Supplement 9 [file Supplemental_Fig_S9.pdf]

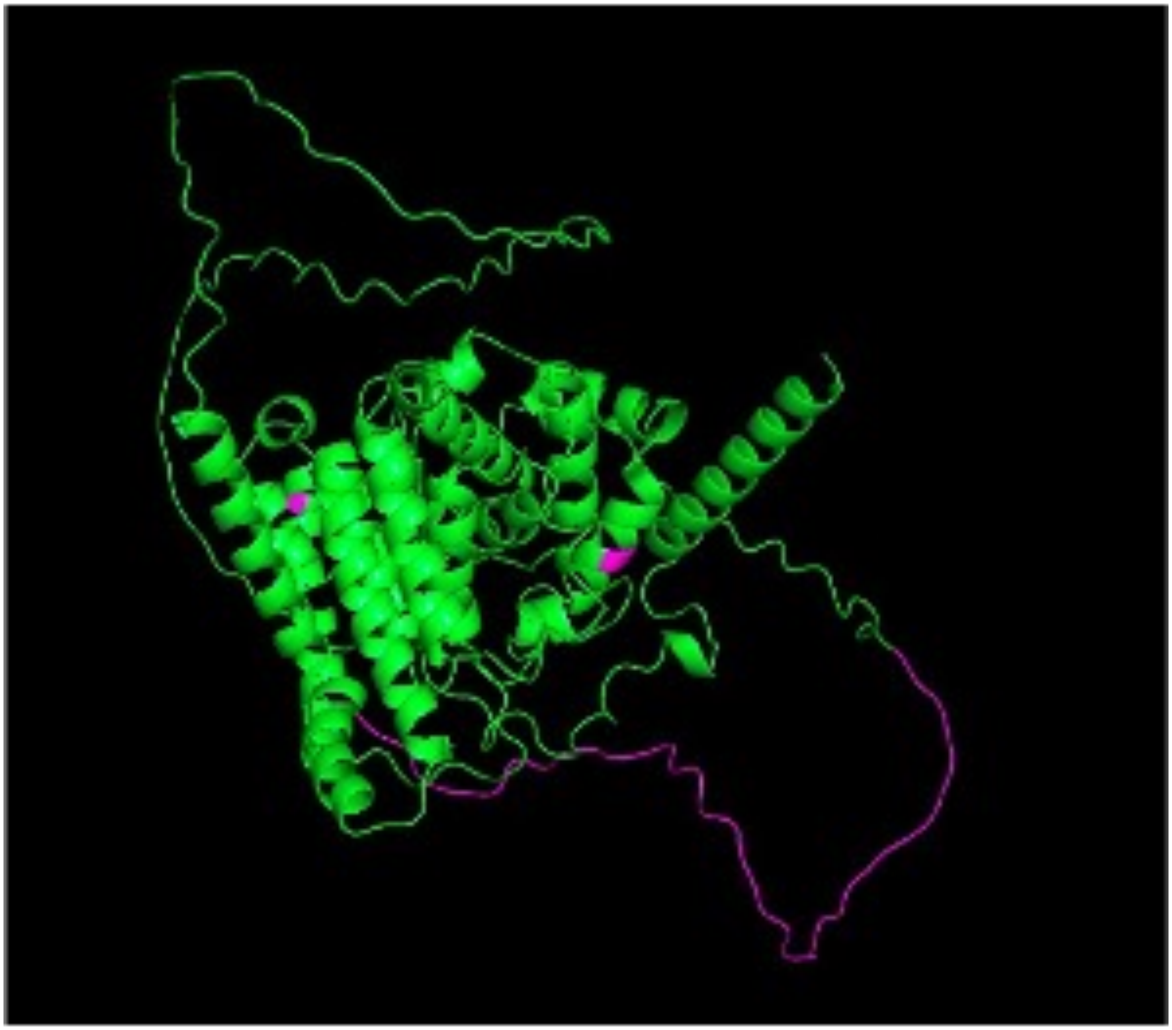

Supplemental Figure S9: Human *TBC1D3* predicted tertiary structure. Human *TBC1D3* was predicted with Alphafold2 (https://alphafold.ebi.ac.uk/). Human lineage amino acid changes, including the modified carboxy terminus, are indicated with violet. We observe that the 41 aa novel C-terminus tertiary structure could not be predicted and is disordered.
