# Supplementary material for: Independent expansion, selection, and hypervariability of the TBC1D3 gene family in humans
Source: Genome Res. 2024 Nov;34(11):1798–810. doi: 10.1101/gr.279299.124 (PMC11610581; doi:10.1101/gr.279299.124)
Supplement: Supplement 10 [file Supplemental_Fig_S10.pdf]

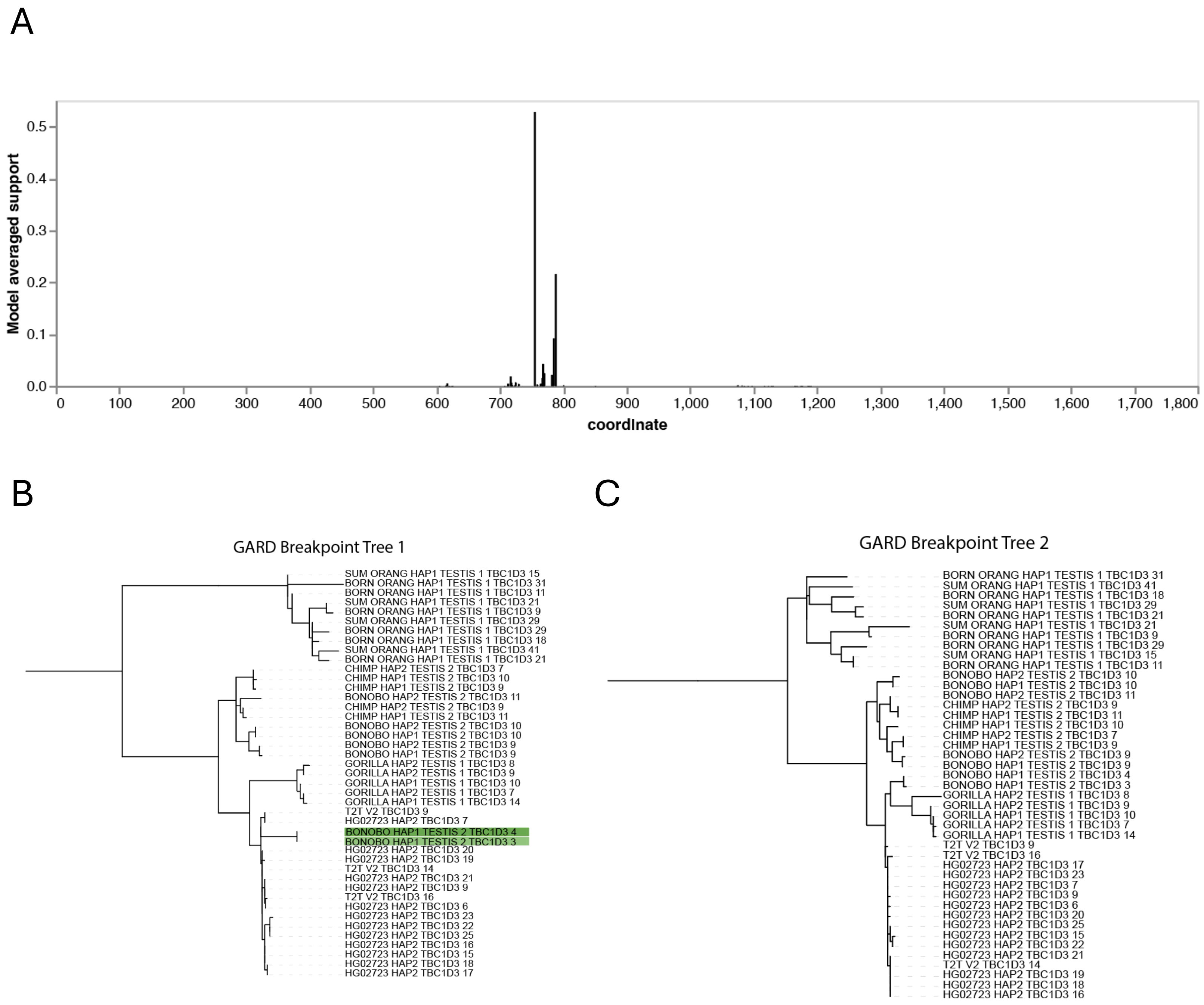

Supplemental Figure S10: Recombination within *TBC1D3* gene model. GARD was run on the codon aligned MSA of expressed *TBC1D3* paralogs to investigate if sites under selection or lineage specific expansions may be explained by gene conversion. **(A)** GARD identified evidence for one breakpoint *TBC1D3* bp 755, corresponding to AA 252, and segmenting the multiple sequence alignment in two subsequences. **(B)** Inferred phylogeny of subsequence 1, the first 755 bp of *TBC1D3*. Green colored lineages illustrate bonobo paralogs nested among human *TBC1D3* copies. **(C)** Inferred phylogeny for the last 1045 bp of *TBC1D3*. For both (B) and (C), species separation is largely maintained, and the inferred breakpoint is not proximal to any sites identified to be undergoing positive selection.
