# Supplementary material for: Independent expansion, selection, and hypervariability of the TBC1D3 gene family in humans
Source: Genome Res. 2024 Nov;34(11):1798–810. doi: 10.1101/gr.279299.124 (PMC11610581; doi:10.1101/gr.279299.124)
Supplement: Supplement 11 [file Supplemental_Fig_S11.pdf]

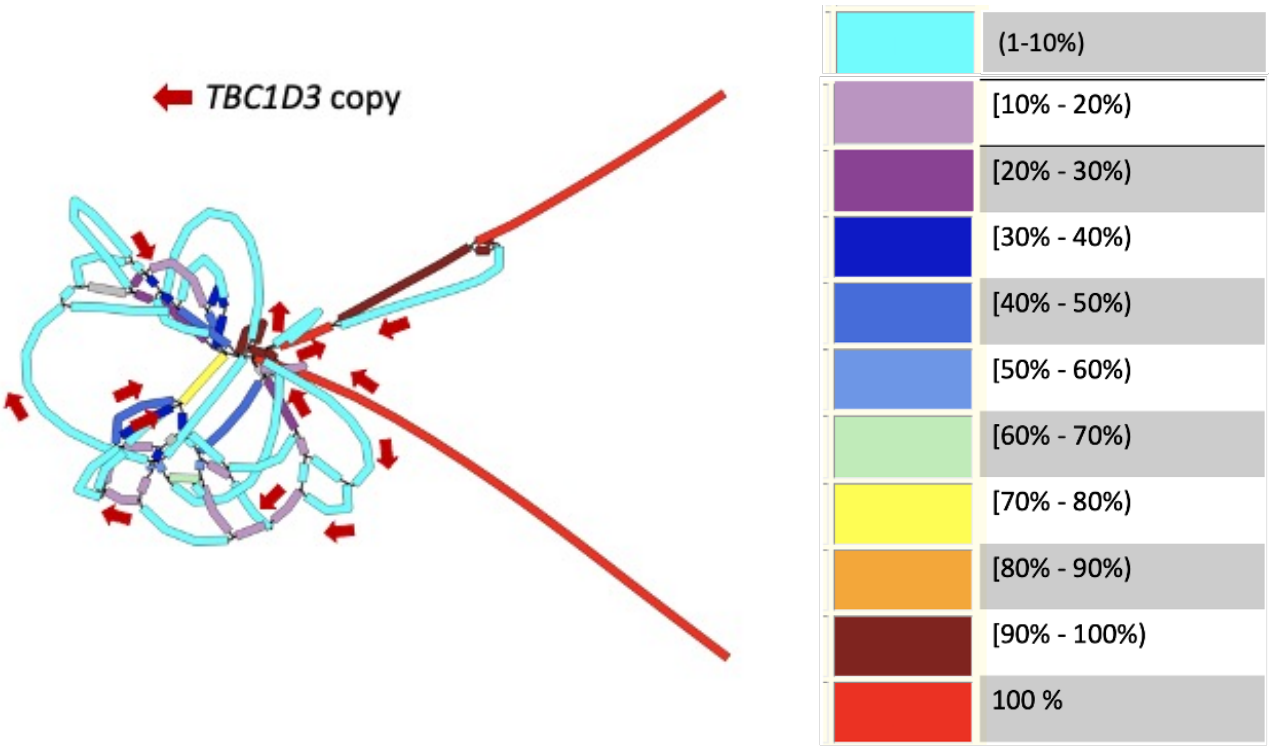

Supplemental Figure S11: Minigraph pangenome graph. We generated a pangenome graph for *TBC1D3* using validated human haplotypes with Minigraph (settings -S -xggs -L 250 -r 100000). Graph segments are colored to represent the proportion of haplotypes that span the given segment, with light red indicating 100% representation and light blue indicating a single-haplotype traversal. *TBC1D3* paralogs are marked with arrows along the graph. We observe that *TBC1D3* structural variation is poorly reduced by Minigraph, where most copies reduce to nodes with single-haplotype support.
