# Supplementary material for: Independent expansion, selection, and hypervariability of the TBC1D3 gene family in humans
Source: Genome Res. 2024 Nov;34(11):1798–810. doi: 10.1101/gr.279299.124 (PMC11610581; doi:10.1101/gr.279299.124)
Supplement: Supplement 12 [file Supplemental_Fig_S12.pdf]

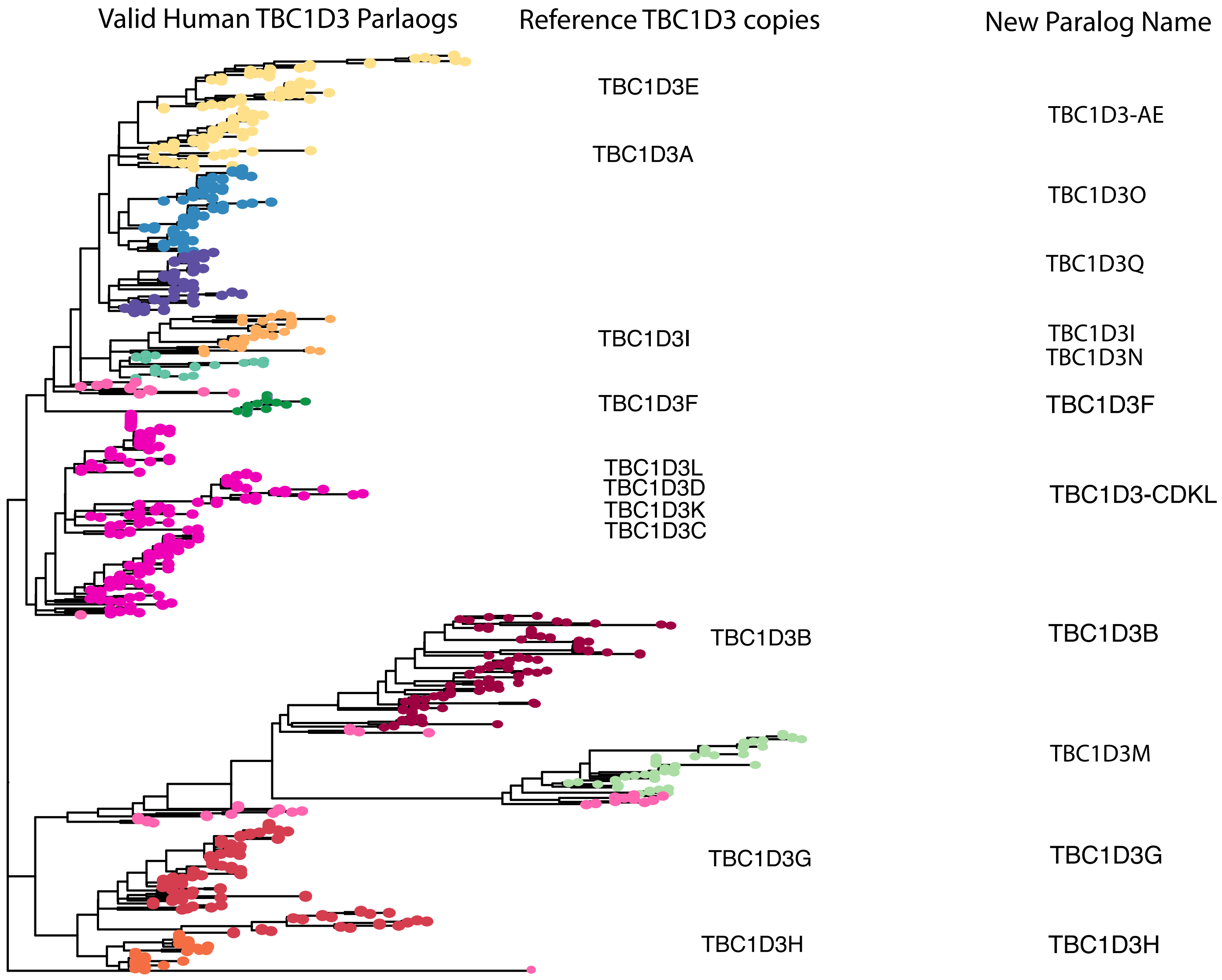

Supplemental Figure S12: Pangenomic clustering and naming. Maximum likelihood phylogeny with ~9600 bp of all human *TBC1D3* cluster 1 and 2 copies, outgrouped to chimpanzee *TBC1D3*. The paralog groups are defined by a heuristic intra-group allelic cutoff based on expected allelic variation in SD sequence (Methods). The first column of labels shows reference GRCh28 paralog locations within the phylogeny. The final column shows the new name given to the associated clusters. Most inherited the name assigned in GRCh28, or a concatenation when multiple paralogs mapped to a common cluster (*TBC1D3-AE; TBC1D3-CDKL*). Four novel population paralogs not included in GRCh38 were identified (*TBC1D3-M,TBC1D3-N, TBC1D3-O, TBC1D3-Q*).
